# Identification and validation of central amygdala FGFR1 as a therapeutic target for alcohol use disorder using single-nucleus sequencing in rats

**DOI:** 10.1038/s41467-026-77302-9

**Published:** 2026-09-02

**Authors:** Estelle Barbier, Leon Höglund, Li Xu, Nihal A. Salem, Elizabeth A. Osterndorff-Kahanek, Riccardo Barchiesi, Antonio Lacorte, Lief E. Fenno, Robert O. Messing, R. Dayne Mayfield, Markus Heilig

**Affiliations:** 1 Center for Social and Affective Neuroscience, BKV, Linköping University, Linköping, Sweden; 2 Waggoner Center for Alcohol and Addiction Research and Departments of Neuroscience and Neurology, University of Texas at Austin, Austin, TX USA; 3 Department of Pharmacy and Biotechnology, Alma Mater Studiorum – University of Bologna, Bologna, Italy; 4 Center for Neuroscience, University of Camerino, Camerino, Italy

**Keywords:** Molecular neuroscience, Addiction

## Abstract

A significant minority of alcohol users develop alcohol addiction, characterized by continued use despite negative consequences, referred to as compulsive-like. We previously showed that vulnerability to compulsive-like alcohol use can be modeled in rats using punished alcohol self-administration and in male rats is mediated by PKCδ+ neurons in the central nucleus of the amygdala (CeA). Here, we used cell-type-specific transcriptomics to identify molecular mechanisms underlying individual differences in this behavior. Transcriptional changes were restricted to a limited number of CeA neuronal populations, including PKCδ+ neurons, where weighted Gene Co-expression Network Analysis identified an upregulated co-expression module in punishment-resistant rats with FGFR1 as a druggable upstream regulator. Selective silencing of *FgfR1* in PKCδ+ neurons normalized elevated PKCδ+ expression, and reduced punishment-resistant alcohol self-administration. This effect was recapitulated by systemic administration of the FgfR1-antagonist PD173074. These findings identify distinct CeA circuits that promote addiction vulnerability, and position FGFR1 as potential therapeutic targets.

## Abstract (web summary)

Compulsive alcohol use is a defining feature of alcohol addiction, but its mechanisms remain unclear. Here, authors show that FGFR1 signaling in central amygdala PKCδ+ neurons promotes compulsive alcohol use and may offer a therapeutic target.

## Introduction

Alcohol use is a major contributor to global disease burden, yet treatments remain limited^1,2^. This highlights an unmet need for mechanistically informed therapeutic strategies. Only a minority of regular drinkers develop addiction [here equated to alcohol dependence according to the WHO ICD-10, or moderate-to-severe alcohol use disorder, AUD, according to the US DSM-5 diagnostic systems, respectively^3,4^], suggesting that vulnerability to alcohol addiction arises from biological mechanisms that differ between people, and remain incompletely understood.

A key diagnostic criterion for alcohol addiction is persistent alcohol use despite adverse consequences, often referred to as compulsive use. This pattern is strongly associated with the transition to addiction and distinguishes people whose drinking becomes problematic from social drinkers whose use remains controlled. Differences in compulsive use can be captured in people under controlled laboratory conditions^5^. Specifically, in a punishment-based fMRI task, social drinkers substantially reduced or ceased their responding for alcohol when responding was associated with a mild electric shock, while heavy drinkers persisted. Punishment resistance in this task was also highly correlated with scores on the clinically validated Obsessive-Compulsive Drinking Scale.

In rats, individual differences in compulsive-like alcohol taking can be modeled using a similar task, in which operant responding for alcohol results in delivery of the alcohol reward, but is also associated with an electric footshock^6–8^. Under these conditions, only a subset of rats continues to self-administer alcohol despite punishment. Convergent neuroimaging and circuit-mapping studies reveal parallel involvement of the anterior insula-nucleus accumbens pathway in punishment-resistant alcohol seeking in both species^5,6^, underscoring the translational relevance of this model.

In addition to cortical circuits^6,9^, the central amygdala (CeA) has emerged as a critical hub for individual differences in alcohol-associated behaviors. Previous work from our group demonstrated that GABAergic activity within CeA biases rats toward choosing alcohol over natural rewards and promotes alcohol self-administration despite punishment^8^. Within this circuit, a population of PKCδ-expressing GABAergic cells plays a causal role in punishment-resistant alcohol self-administration, consistent with known functions of PKCδ+ neurons in emotional regulation, and also with their heightened sensitivity to alcohol withdrawal^10^. However, the molecular mechanisms that result in elevated PKCδ+ neuron activity in the minority of rats that are vulnerable to punishment-resistant alcohol self-administration remain unknown.

To identify these mechanisms, we performed large-scale single-nucleus RNA sequencing (snRNA-seq) of CeA from male and female rats classified as punishment-resistant (compulsive-like, *n* = 24; 12 males / 12 females) or punishment-sensitive (non-compulsive, *n* = 24; 12 males / 12 females), or yoked to the compulsive-like group (*n* = 24; 12 males / 12 females). Whereas prior studies in addiction models have typically relied on significantly smaller numbers of animals per condition, this scale enabled robust cell-type resolution and subclustering, and provided a transcriptomic resource for exploring CeA dysregulation behind punishment-resistant alcohol self-administration.

Although our snRNA-seq discovery effort was performed in both male and female rats, prior work as well as ongoing experiments indicate fundamental sex differences in mechanisms associated with punishment-resistant alcohol self-administration^11^. In males, this phenotype is associated with alcohol-reward sensitivity and differential PKCδ expression, whereas these relationships are absent in females^8^. Given these differences, the present study focused on identifying and functionally validating male-specific mechanisms, while investigations into female-specific pathways are ongoing.

Our analysis in male rats identified a limited set of distinct transcriptomic signatures associated with punishment-resistant alcohol self-administration. One of these was enriched in PKCδ+ neurons previously linked to this behavior. Among the top driver genes of this differentially expressed module was Fibroblast Growth Factor Receptor 1 (FgfR1). We confirmed the selective overexpression of this transcript in PKCδ+ neurons using RNAscope in situ hybridization, and used a PKCδ-Cre rat line^12^ to selectively knock down FgfR1 within this population. The knockdown reduced *Prkcd* mRNA expression and decreased punishment-resistant but not unpunished alcohol self-administration. A behaviorally specific and dose-dependent suppression of punishment-resistant self-administration was also seen following systemic administration of the FgfR1 antagonist PD173074.

Collectively, these findings identify FfgR1 signaling in PKCδ + CeA neurons as a key molecular driver of compulsive-like alcohol use. This suggests that FgfR1 or signaling molecules downstream of it may offer therapeutic targets for reducing alcohol use despite negative consequences in men.

## Results

### A subset of outbred rats exhibits compulsive-like alcohol self-administration

To characterize transcriptomic signatures associated with punishment-resistant alcohol self-administration, rats were trained to self-administer alcohol and then exposed to a punishment phase in which alcohol delivery was paired with a mild foot shock. Individual resistance to punishment was quantified using a resistance score (RS) [see “Methods”^8^;], which compares each rat’s responding during punished sessions to its own baseline responding during unpunished sessions. This normalization accounts for variability in baseline operant responding prior to the introduction of punishment, providing a measure of punishment resistance that specifically reflects behavioral changes induced by foot shock. When behavior is not affected, the RS = 0.5.

**Fig. 1:**
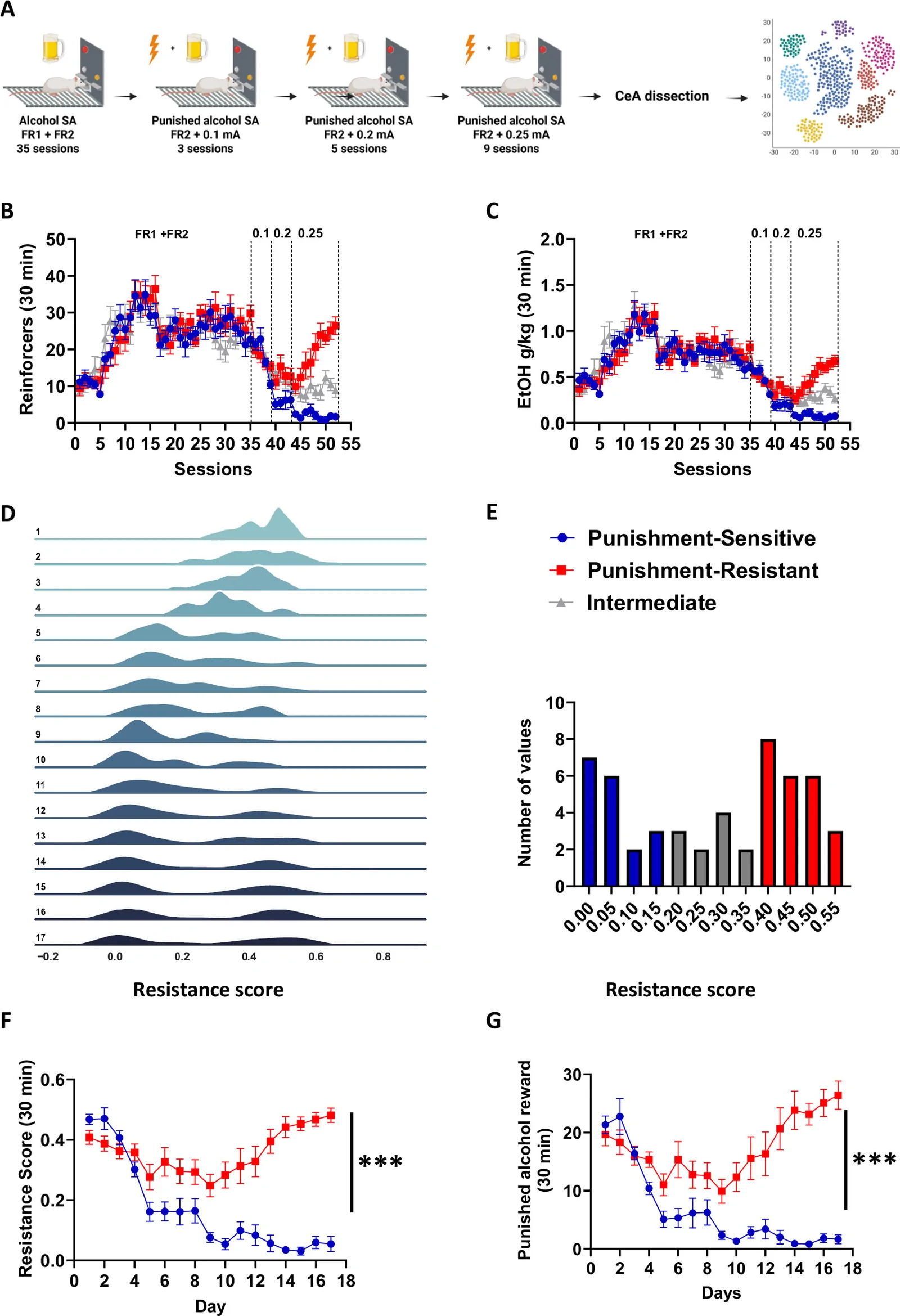
Description of the behavioral model. **A** An experimental timeline. **B** Alcohol self-administration (reinforcers) across all behavioral phases (Punishment-sensitive in blue *n* = 12; punishment-resistant in red *n* = 12; intermediate in gray *n* = 14; alcohol self-administration for punishment-sensitive and punishment-resistant are shown only for animals used for snRNA sequencing). Error bars indicate SEM. **C** Alcohol intake in g/kg across all behavioral phases (Punishment-sensitive in blue *n* = 12; punishment-resistant in red *n* = 12; intermediate in gray *n* = 14; alcohol intake for punishment-sensitive and punishment-resistant are shown only for animals used for snRNA sequencing). Error bars indicate SEM. **D** The probability density of resistance score over the course of punished (compulsive-like) self-administration shows the emergence of two distinct populations. The density plot includes animals classified as either punishment-sensitive or punishment-resistant (*n* = 24). **E** The average of the last 3 days’ resistance scores shows a bimodal distribution for animals used for snRNA sequencing (red and blue; punished-resistant and punished sensitive, respectively; gray bars show “intermediate” rats). **F**, **G** Resistance score and punished alcohol reward averages for rats used for snRNA sequencing. **F** Resistance score across punishment sessions in punishment-resistant (red, *n* = 12) and punishment-sensitive (blue, *n* = 12) rats. A linear mixed-effects model with group, session, and their interaction as fixed effects, rat as a random intercept, and an AR(1) covariance structure revealed a significant effect of group (F_(1,94.5)_ = 39.3, *P* = 1.0795 × 10^−8^). Error bars indicate SEM. **G** Number of punished alcohol rewards earned across punishment sessions in punishment-resistant (red, *n* = 12) and punishment-sensitive (blue, *n* = 12) rats. A linear mixed-effects model with the same specification revealed a significant effect of group (F_(1,93.7)_ = 29.8, *P* = 3.84 × 10^−7^). ***P < 0.001 indicates a significant main effect of group in F and G. Panel 1A is created in BioRender (Johansson A, 2026; https://BioRender.com/ug9kewr), and licensed under CC BY 4.0.

Consistent with previous findings^8^, the distribution of resistance scores was initially unimodal but evolved into a bimodal pattern across punished sessions, indicating the emergence of two distinct behavioral phenotypes: punishment-resistant (compulsive-like) and punishment-sensitive (non-compulsive Fig. 1). Punishment-sensitive rats showed marked suppression of punished self-administration (Fig. 1F; RS ∼ 0.08, ∼ 84% decrease; Fig. 1G; Rewards ∼ 2). In contrast, punishment-resistant rats maintained high levels of responding despite foot shock (Fig. 1F; RS ∼ 0.46, ∼ 18% decrease; Fig. 1G; Reward ∼ 25). In this procedure, 25 rewards correspond to modest blood alcohol concentrations (BACs ∼ 20 mg/dl^8^, allowing us to primarily study molecular mechanisms behind persistence of the motivation for alcohol seeking and taking despite negative outcomes, rather than transcriptomic changes driven by pharmacological effects of alcohol.

### Cell-type clustering

We next performed single-nucleus RNA sequencing of the CeA in punishment-resistant and punishment-sensitive rats. To control for transcriptional changes induced by foot shock alone, we also included a yoked control group of alcohol-naïve rats; these received the same number of foot shocks as the punishment-resistant rats but did not have access to alcohol self-administration.

**Fig. 2:**
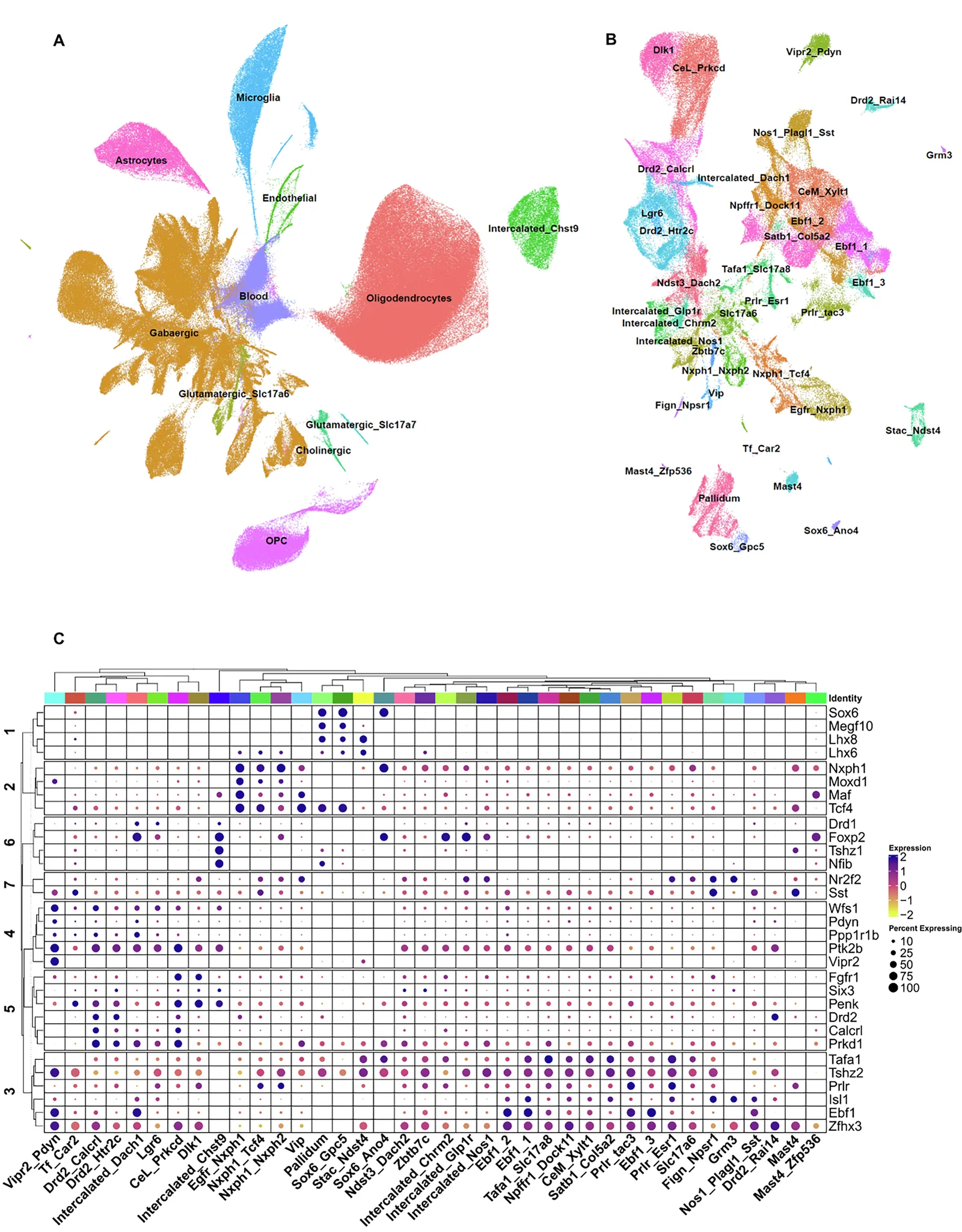
Cell type identification in the CeA + sub-clustering. **A** UMAP of cells from the CeA, with clusters corresponding to cell types based on expression of known markers. **B** UMAP of inhibitory subtypes in the CeA, with labels showing unique markers of each subcluster. **C** Hierarchical clustering of the expression of genes was used to identify groups of inhibitory subclusters.

After normalization and dimensionality reduction, Louvain clustering identified 18 distinct clusters, annotated by known marker genes and merged into larger main cell types^10,13,14^ (Fig. 2A). Expected cell types were identified, including oligodendrocytes, glutamatergic, GABAergic and cholinergic neuronal subtypes, astrocytes, microglia, endothelial cells and oligodendrocyte precursor cells. In line with the known cellular composition of the CeA, oligodendrocytes were the most abundant glial population (150,768 nuclei), while GABAergic neurons constituted the predominant neuronal cell type (138,267 nuclei).

Building on our previous work, we focused on GABAergic neurons, which we have shown to play a key role in individual vulnerability to punishment-resistant alcohol self-administration^8^ and in promoting choice of alcohol over non-drug rewards^15^. Within the CeA, GABAergic neurons form a heterogeneous population with distinct functional roles^10,16^, suggesting that specific subtypes may differentially contribute to maladaptive alcohol-associated behaviors. To uncover the molecular basis of these behavioral differences, we performed subclustering of CeA GABAergic neurons, enabling us to identify subtype-specific transcriptomic signatures associated with punishment-resistant alcohol intake.

Using the same pipeline as for the main cell populations, we identified 40 distinct subclusters, which, after annotation, were merged into 38 subtypes. Subcluster annotation was performed based on known marker genes supported by differential expression analysis between clusters (Fig. 2B). Hierarchical clustering of annotated GABAergic subtypes, based on genes previously reported to exhibit region-specific enrichment within the CeA, showed the expected regional distinctions (Fig. 2C; see supplementary section 1).

### Punishment-resistant alcohol self-administration is associated with gene expression changes within PKCδ+ neurons

To identify transcriptomic signatures of distinct GABAergic subtypes in the CeA associated with punishment-resistant alcohol self-administration, we used high-dimensional weighted gene co-expression network analysis (hdWGCNA). This approach surpasses conventional differential gene expression analysis by enabling the identification of cell–type–specific gene networks^17^. Furthermore, it facilitates the detection of putative regulatory hub genes exerting coordinated control over the expression of other genes, potentially providing mechanistic insights into transcriptional regulation within each subtype^18^. As described in “Methods”, a consensus analysis was conducted to identify co-expression networks conserved across the three experimental groups: punishment-resistant, punishment-sensitive, and yoked footshock-exposed controls.

**Fig. 3:**
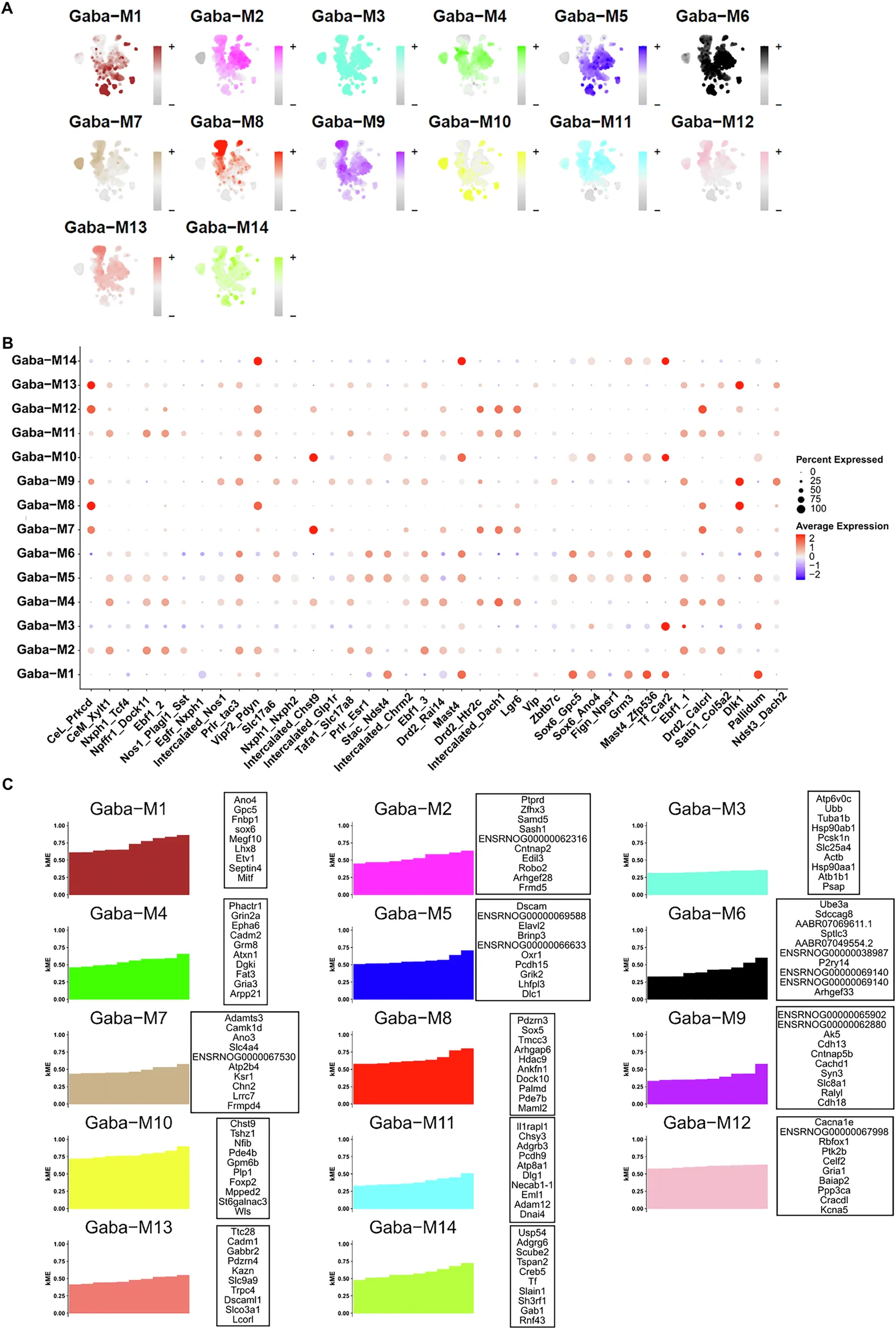
Description of the hdWGCNA analysis. **A** UMAPs showing the expression of each module’s eigengene. **B** Expression of module eigengenes in GABAergic subtypes. **C** Top 10 genes of each module based on connectivity (kME).

The network construction generated 14 co-expression modules with varying levels of cell type specificity and eigengene-based connectivity (kME) (Fig. 3A–C). For example, module 3 exhibited relatively uniform expression across all GABAergic cell subtypes, and its constituent genes showed comparatively low kME values (Fig. 3C), suggesting that this module likely reflects broad, general biological processes rather than cell–type–specific transcriptional programs. This interpretation was further supported by pathway enrichment analysis, in which module 3 was enriched for mitochondrial electron transport and ATP production, core metabolic processes essential for cellular energy homeostasis and generally conserved across cell types (Fig. 3C). In contrast, module 8 was expressed mainly in *PKCδ*^+^ and *Dlk1*^+^ neurons, with its genes showing higher kME values, suggesting a strong, cell type-specific gene network signature (Fig. 3C).

**Fig. 4:**
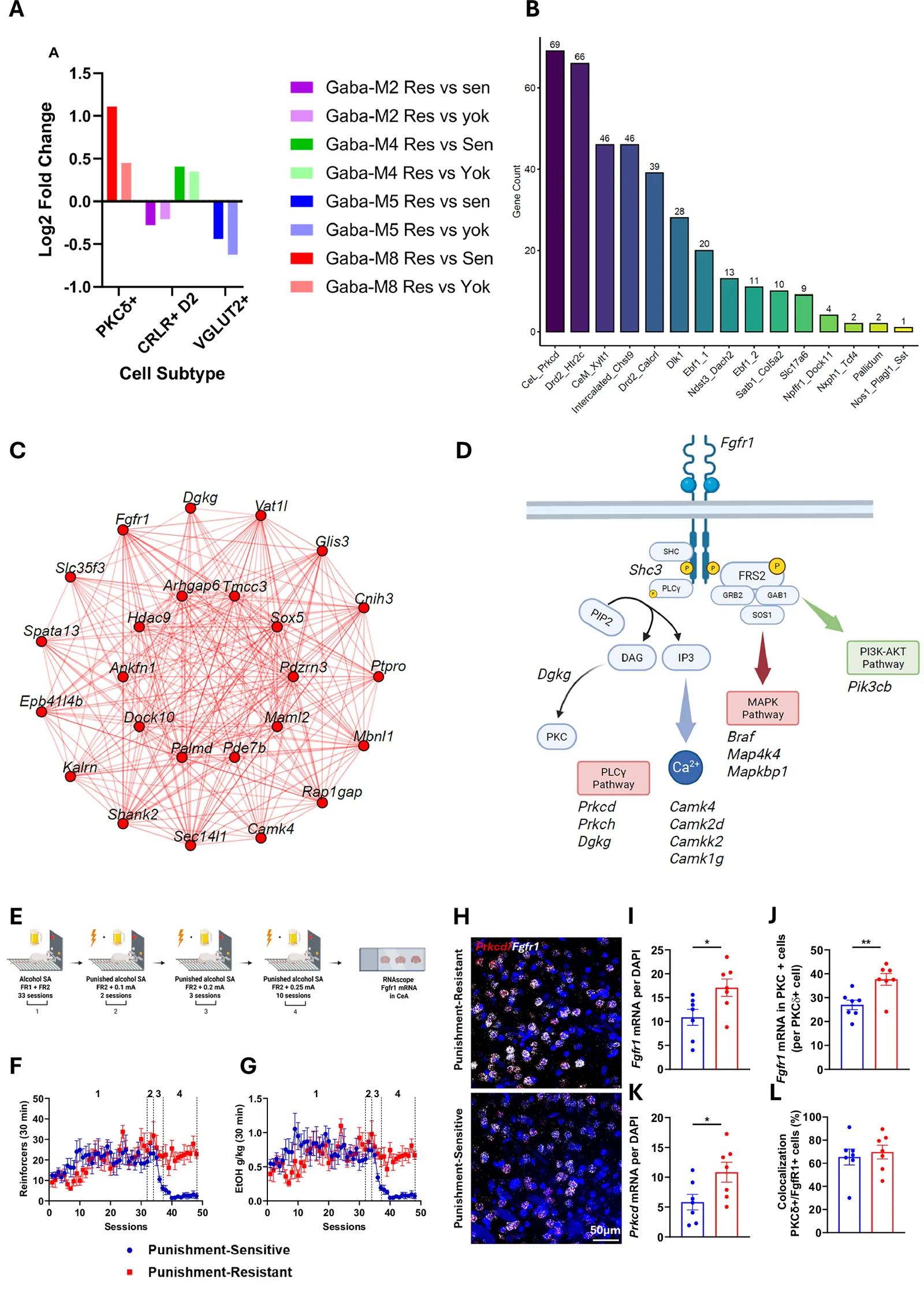
Differential expression of gene modules among three cell types: CRLR + D2-neurons, VGLUT2+ neurons and PKCδ+ expressing neurons. **A** Four co-expression module eigengenes were differentially expressed in compulsive-like rats compared to both non-compulsive rats and foot-shock yoked controls, adjusted *P* value < 0.05 (exact adjusted *P* values from left to right in bargraph; 2 × 10^−6^, 0.04, 0.0004, 0.0001, 4.92 × 10^−10^, 2.78 × 10^−7^, 0.01, 0.004). Res vs Sen = Punishment-resistant vs Punishment-sensitive. Res vs Yok = Punishment-resistant vs foot shock-yoked control. Harmonized module eigengenes were compared between conditions using a two-sided Wilcoxon rank-sum test. *P* values were adjusted for multiple comparisons using the Bonferroni method. **B** Amount of significantly differentially expressed genes per GABAergic subtype. The distinct method compares the full gene-expression distributions between experimental groups using hierarchical, non-parametric permutation tests applied to the sample-level empirical cumulative distribution functions. Benjamini–Hochberg-adjusted *P* values were calculated both within each cell subtype (locally) and across all gene–cell-subtype tests (globally). Genes with globally adjusted *P* < 0.05 were considered statically significant. **C** Top 25 genes based on connectivity in module 8. **D** Signaling pathways downstream of activated *Fgfr1*. Genes in cursive are present in module 8. **E** Experimental timeline. **F** Alcohol self-administration (reinforcers) across all behavioral phases in groups used for RNAscope confirmation of differential *FgfR1* expression (punishment-sensitive in blue *n* = 7; punishment-resistant in red *n* = 7). Error bars indicate SEM. **G** Alcohol intake in g/kg across all behavioral phases in groups used for RNAscope confirmation of differential FgfR1 expression (punishment-sensitive in blue; *n* = 7; punishment-resistant in red; *n* = 7). Error bars indicate SEM. **H** Representative RNAscope images for *Prkcd* (in red) and *FgfR1* (in white) mRNA expression in CeA of punished-resistant and punished-sensitive rats. **I** *FgfR1* mRNA expression per DAPI+ cells is increased in punishment-resistant (blue; *n* = 7) compared to punishment-sensitive (red; *n* = 7) rats. Data are presented as mean ± SEM. An independent-samples, two-tailed t-test revealed significantly higher *FgFr1* mRNA expression per DAPI+ cell in punishment-resistant rats than in punishment-sensitive rats (t(12) = 2.52, *P* = 0.027). **J** *FgfR1* mRNA expression per PKCδ+ cells. RNAscope data confirmed increased expression of *Fgfr1* in PKCδ+ cells of punishment-resistant (red; *n* = 7) compared to punishment-sensitive (red; *n* = 7) rats (independent-samples t-test (two-tailed), t(12) = 3.38; *P* = 0.0055). Error bars indicate SEM. **K** *Prkcd* mRNA expression per DAPI+ cell in the central amygdala of punishment-sensitive (blue, *n* = 7) and punishment-resistant (red, *n* = 7) rats. Data are presented as mean ± SEM. An independent-samples, two-tailed t-test showed significantly higher *Prkcd* mRNA expression per DAPI+ cell in punishment-resistant rats than in punishment-sensitive rats (t(12) = 2.37, *P* = 0.035). *L* colocalization *Prkcd/FgfR1* (punishment-sensitive in blue; *n* = 7; punishment-resistant in red; *n* = 7). Error bars indicate SEM. * *P* < 0.05; **P < 0.01. Panels 4 C and E are created in BioRender (4 D: Johansson A, 2026; https://BioRender.com/jszncjh; 4 E: Johansson A, 2026, https://BioRender.com/aoe1mvl) and licensed under CC BY 4.0.

We next conducted differential expression analysis of module eigengenes, comparing punishment-resistant rats with both punishment-sensitive rats and yoked controls. Four modules were differentially expressed with the same directionality across both comparisons, indicating that these changes are specific to the punishment-resistant phenotype, rather than being a consequence of exposure to footshocks (Fig. 4A).

We selected module 8 for further investigation based on several considerations. First, this module showed relatively specific expression in well-characterized neuronal subtypes, including *Dlk1*^+^ and *PKCδ*^+^ neurons, making it a compelling candidate for cell type-specific interventions (Fig. 3B). Although module 8 was highly expressed in both populations, differential expression was restricted to *PKCδ*^+^ neurons, where this module was significantly upregulated in rats exhibiting punishment-resistant alcohol self-administration (Fig. 4A). This aligns with our previous work implicating *PKCδ*^+^ neurons in punishment-resistant alcohol self-administration^8^. Further supporting this, transcript-level differential expression analysis demonstrated that PKCδ⁺ neurons harbored the largest number of differentially expressed genes among all GABAergic subtypes (Fig. 4B). Second, module 8 included multiple transcripts encoding proteins previously implicated in addiction-related behaviors and targeted by pharmacological inhibitors, such as *FgfR1* and *Pde7b*^19,20^, underscoring its translational potential (Fig. 4C-D). Third, top Gene Ontology (GO) terms associated with the module, such as calcium-dependent protein kinase activity (Supplementary Fig. S4D), suggest that elevated module 8 activity may be associated with heightened neural activity, a phenomenon our laboratory has previously observed in PKCδ⁺ neurons during punishment-resistant alcohol self-administration^8^. Collectively, these features provided the rationale for our focused analysis of module 8.

Module 8 included key regulators of synaptic plasticity and neuronal signaling, including *Pde7b*, *Hdac9*, *Camk4*, and *FgfR1*^21^. The products of these genes modulate intracellular signaling cascades, transcriptional regulation, and calcium-dependent processes that are fundamental to neuronal excitability and adaptation^22–24^. Through these molecular functions, module 8 may contribute to elevated activity of *PKCδ*^*+*^ neurons, a causal mechanism contributing to punishment-resistant alcohol self-administration^8^.

Having identified module 8 as a candidate driver of punishment-resistant alcohol self-administration, we next sought to identify genes within this module with the potential to (1) modulate PKCδ^+^ cell activity, (2) regulate module function, and (3) represent potential therapeutic targets with translational relevance. We prioritized genes exhibiting enriched expression in PKCδ^+^ cells relative to other cell types. Although the 25 genes with the highest kME values in module 8 were highly expressed in PKCδ^+^ cells, several displayed particularly strong specificity for PKCδ^+^, including *Fgfr1, Pdzrn3*, *Palmd*, and *Glis3* (Supplementary Fig. S5).

To assess the functional relevance of these candidates, we performed pathway analysis on module 8 genes, which revealed significant enrichment (*P* < 0.01) of multiple canonical signaling pathways, including RHO GTPase cycle, synaptogenesis signaling pathway, DAG and IP₃ signaling, calcium signaling, and the RAF/MAP kinase cascade, processes known to occur downstream of activated FGFR1^25,26^ (Supplementary Data 1). In parallel, pathway analysis of differentially expressed genes in PKCδ⁺ neurons derived from the same snRNA-seq dataset showed significant enrichment and predicted activation of synaptogenesis and the RAF/MAP kinase signaling in compulsive-like rats (Supplementary Data 2). Together, these analyses provide convergent evidence that altered FGFR1 activity in PKCδ⁺ neurons may contribute to punishment-resistant alcohol self-administration.

Consistent with this pathway-level convergence, several established downstream effectors of FGFR1 signaling were represented among the genes comprising module 8, supporting the interpretation that FGFR1-associated signaling contributes to the transcriptional state observed in CeA *PKCδ+* neurons (Fig. 4D). These effectors include *Shc3*, a predicted signaling adapter that couples activated growth factor receptors to downstream pathways; components of the RAF/MAPK (ERK) cascade, including *Braf, Map4k4, Mapkbp1*, which regulate long-term changes in neuronal activity, synaptic plasticity, and gene expression; and the PI3K/AKT pathway component *Pik3cb*, which promotes neuronal survival, growth, and plasticity^22^.

In addition, several components of the PLCγ pathway, which relays calcium-mediated signals downstream of *FgfR1,* were also present in the module. These included *Prkch, Prkcd* and *Dgkg*, as well as calcium-dependent effectors such as *Camk4, Camk2d, Camkk2 and Camk1g*^27,28^. Together, these findings indicate that multiple canonical FGFR1 downstream signaling branches (MAPK, PI3K/AKT, and PLCγ) are embedded within the transcriptional architecture of module 8. The convergence of cell-type specific transcriptional changes of FGFR1 and module-level enrichment of its downstream effectors positions FGFR1 as a molecular node linking module 8 to the heightened activity of PKCδ⁺ neurons associated with punishment-resistant alcohol self-administration.

This interpretation is further supported by prior evidence implicating FGFR1 in alcohol-related behaviors. Both systemic and dorsomedial striatum-specific inhibition of FGFR1 reduces alcohol consumption and preference, whereas infusion of its ligand FGF2 into the dorsomedial striatum increases alcohol intake^20,29^. Moreover, FGF2 expression is upregulated in CeA astrocytes during alcohol withdrawal, coinciding with increased alcohol self-administration^10^, suggesting that FGFR1 signaling contributes to alcohol-induced neuroadaptations.

### Punishment-resistant alcohol self-administration is modulated by FGFR1 in PKCδ^+^ CeA neurons

Because FGFR1 emerged as a prominent network node in our dataset and is a pharmacologically tractable target, we investigated its role in PKCδ⁺ neurons in punishment-resistant alcohol self-administration.

RNAscope in situ hybridization validated our snRNA-seq findings, showing robust co-localization of *FgfR1* with *PKCδ+* neurons and a significant upregulation of *FgfR1* mRNA in *PKCδ+* neurons of punishment-resistant rats (Fig. 4J; independent-samples t-test (two-tailed), t(12) = 3.38; *P* = 0.0055). Because differences in brain alcohol exposure, in addition to compulsivity-related mechanisms, might influence *FgfR1*, individual-level alcohol intake was included in the analysis. When controlling for alcohol exposure, *FgfR1* mRNA expression was robustly increased in punished-resistant compared to punished-sensitive rats (ANCOVA: significant main effect of group: F_(1,11)_ = 24.32, *P* = 0.0004). There was also a significant main effect of alcohol intake F_(1,11)_ = 11.55, *P* = 0.006). In a replication of our previous findings^8^, *Prkcd* mRNA expression was also elevated in punishment-resistant compared with punishment-sensitive rats (one-way ANOVA: F_(1,12)_ = 7.3, *P* = 0.02; Fig. 4K).

**Fig. 5:**
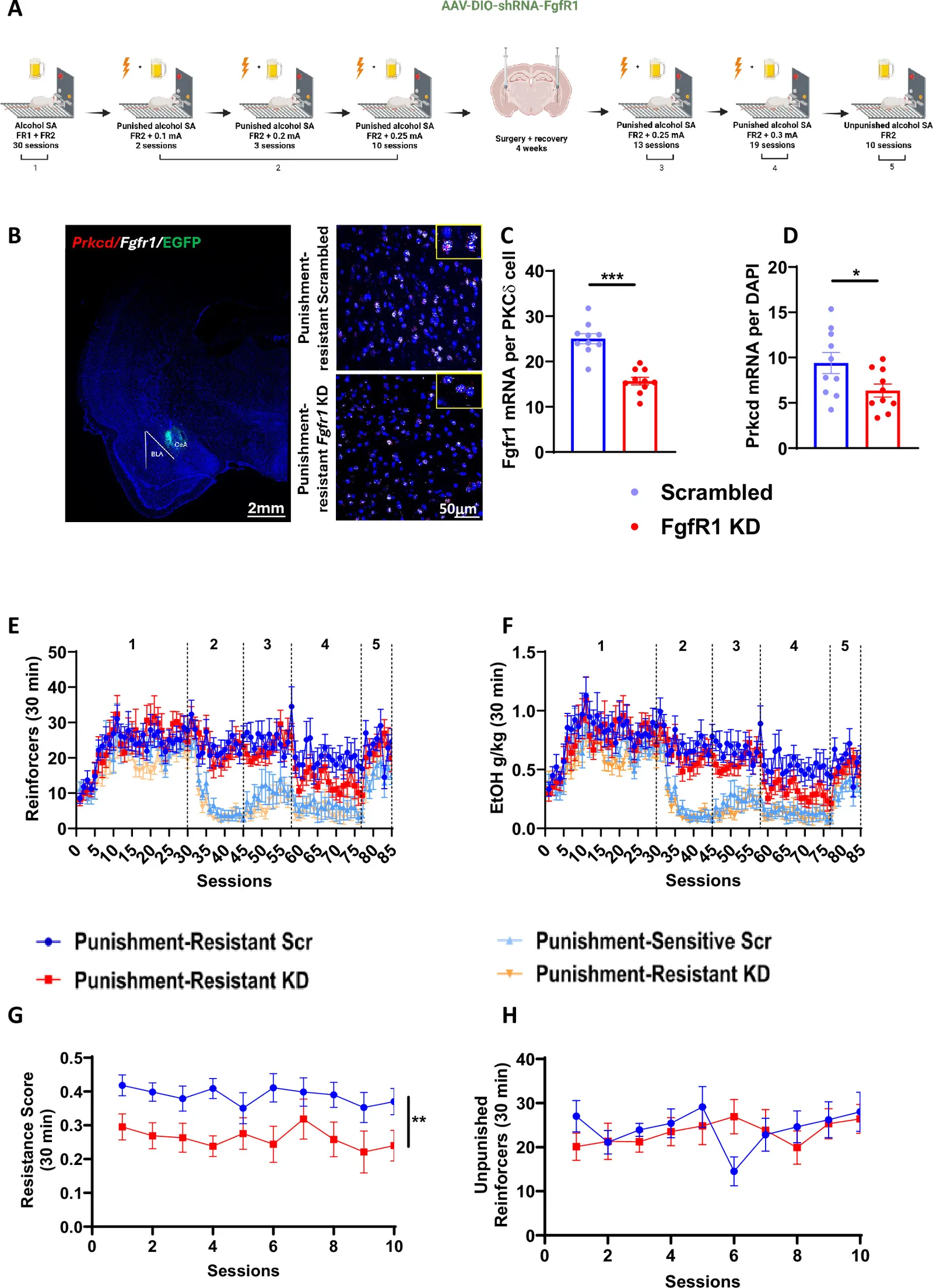
Punishment-resistant alcohol self-administration is modulated by FGFR1 in PKCδ + CeA neurons. **A** Experimental timeline. **B** After punished self-administration training, rats received a bilateral infusion of a Cre-dependent AAV vector encoding either a shRNA targeting *Fgfr1* (in red) or a scrambled control (in blue) into the CeA. **C** RNAscope data confirming successful knockdown of *Fgfr1* mRNA in PKCδ cells (scrambled in blue; *n* = 10; *FgfR1* KD in red; *n* = 10; two-tailed *t*-test, t(18) = 6.70; *P* = 0.000003). Error bars indicate SEM. **D** RNAscope analysis also shows a significant decrease in *Prkcd* mRNA in *FgfR1KD* compared to scrambled rats (scrambled in blue; *n* = 10; *FgfR1* KD in red; *n* = 10; two-tailed *t*-test, t(18) = 2.24; *P* = 0.038). Error bars indicate SEM. **E** Alcohol self-administration (reinforcers) across all behavioral phases (punishment-sensitive Scr in light blue; *n* = 10; punishment-sensitive KD in orange; *n* = 10; Punishment-resistant Scr in blue; *n* = 10; Punishment-resistant KD in red; *n* = 10). Error bars indicate SEM. **F** Alcohol intake in g/kg across all behavioral phases (punishment-sensitive Scr in light blue; *n* = 10; punishment-sensitive KD in orange; *n* = 10; Punishment-resistant Scr in blue; *n* = 10; Punishment-resistant KD in red; *n* = 10). Error bars indicate SEM. **G** Knockdown of *Fgfr1* significantly decreased compulsive-like self-administration (resistance score; Punishment-resistant Scr in blue; *n* = 10; Punishment-resistant KD in red; *n* = 10; RM ANOVA main effect of group; F_(1:162)_ = 7.9; *P* = 0.01). Error bars indicate SEM. A similar result was obtained when analyzing untransformed numbers of rewards obtained (supplementary Fig. S7); **H** knockdown of *Fgfr1* did not affect unpunished alcohol self-administration (Punishment-resistant Scr in blue; *n* = 10; Punishment-resistant KD in red; *n* = 10). Error bars indicate SEM. KD knockdown; Scr Scrambled control. Panel 5A is created in BioRender (Johansson A, 2026; https://BioRender.com/otdv8hn) and licensed under CC BY 4.0.

To directly test the causal role of FGFR1 in *PKCδ+* neurons for compulsive-like alcohol self-administration, we used a Cre-dependent viral strategy in a *PKCδ-Cre* rat line^12^, to selectively reduce *FgfR1* expression in this neuronal population (Fig. 5A). Bilateral CeA infusion of a Cre-dependent AAV vector encoding an *shRNA* targeting *FgfR1* produced an approximately 40% reduction in *FgfR1* mRNA within PKCδ⁺ neurons (one-way ANOVA: F_(1,18)_ = 44.8; *P* < 0.001 Fig. 5B, C). Of note, *FgfR1* knockdown also significantly reduced *Prkcd* mRNA abundance in punishment-resistant rats, suggesting a reversal of the elevation found in this population, and supporting the conclusion that FGFR1 knockdown effectively attenuates downstream PLCγ-mediated signaling (Fig. 5D; one-way ANOVA: F_(1,18)_ = 4.9; *P* = 0.04).

Functionally, *FgfR1* knockdown significantly reduced punished alcohol self-administration, resulting in lower resistance scores in *FgfR1* knockdown rats compared with scrambled controls (Fig. 5G; RM ANOVA main effect of group; F_(1:162)_ = 7.9; *P* = 0.01). This effect was also seen when analyzing untransformed numbers of rewards earned (supplementary Fig. S7). This effect was specific to punished self-administration and did not alter unpunished alcohol self-administration (Fig. 5H). In addition, *FgfR1* KD did not affect breakpoint responding, indicating that decreased punished alcohol self-administration was not secondary to decreased motivation for alcohol (Supplementary Fig. S8; one-way ANOVA main effect of groups; F_(1,18)_ = 1.28; *P* > 0.5). Control experiments further confirmed the specificity of the behavioral effect, as *FgfR1* knockdown did not affect footshock sensitivity (Supplementary Fig. S9; RM ANOVA: F_(1,54)_ = 0.4; *P* = 0.75).

**Fig. 6:**
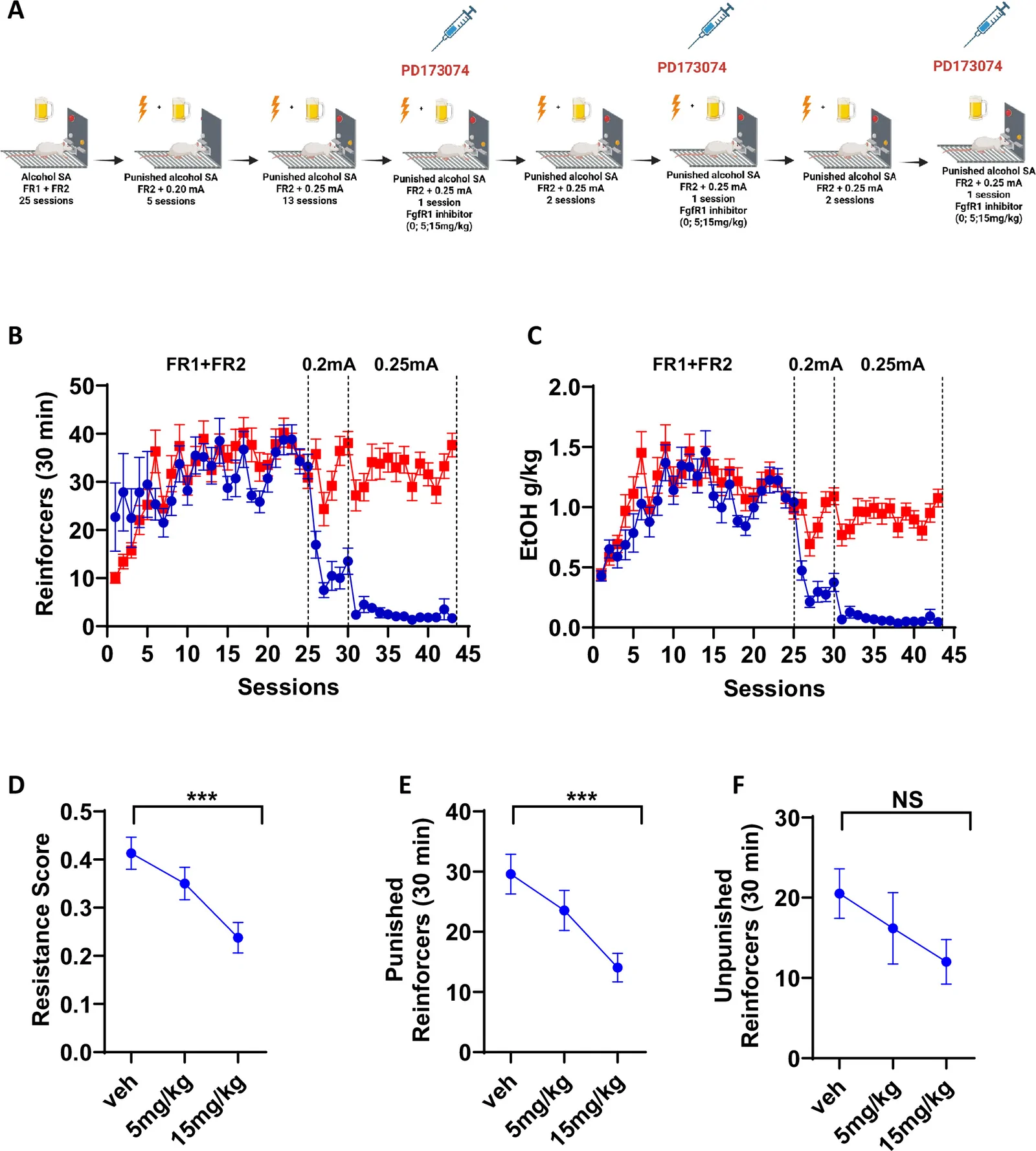
PD173074 dose-dependently suppressed punished alcohol self-administration. **A** Experimental timeline (*n* = 22 punishment-resistant in red; 15 punishment-sensitive in blue). **B** Alcohol self-administration (reinforcers) across all behavioral phases. Error bars indicate SEM. **C** Alcohol intake in g/kg across all behavioral phases (Punishment-sensitive in blue; *n* = 15; Punishment-resistant in red; *n* = 22). Error bars indicate SEM. **D** Systemic (i.p) administration of the selective FGFR1 antagonist PD173074 robustly and dose-dependently suppressed resistance score in punishment-resistant rats (Latin square; vehicle: *n* = 22; 5 mg/kg: *n* = 22; 15 mg/kg: *n* = 22). Repeated measure ANOVA showed a significant main effect of drug (F_(1,42)_ = 10.2; *P* = 0.0002; Newman-Keuls post hoc analysis: 5 mg/kg vs vehicle: *P* = 0.11; 15 mg/kg vs. vehicle: *P* = 0.0002). Error bars indicate SEM. **E** Systemic (i.p) administration of the selective FGFR1 antagonist PD173074 robustly and dose-dependently suppressed punished alcohol self-administration in punishment-resistant rats (Latin square; vehicle: *n* = 22; 5 mg/kg: *n* = 22; 15 mg/kg: *n* = 22). Repeated measure ANOVA showed a significant main effect of drug (F_(1,42)_ = 11.29; *P* = 0.0001; Newman-Keuls post hoc analysis: 5 mg/kg vs vehicle: *P* = 0.17; 15 mg/kg vs. vehicle: *P* = 0.0002). Error bars indicate SEM. **F** The effect of PD173074 on unpunished alcohol self-administration (vehicle: *n* = 10; 5 mg/kg: *n* = 10; 15 mg/kg: *n* = 10). One-way ANOVA showed no significant main effect of drug (F(_1,27)_ = 1.45; *P* > 0.05). Error bars indicate SEM. At these doses, PD173074 did not suppress responding for a non-drug reward, a palatable saccharin solution (see Supplementary Fig. 10). Veh: vehicle. Panel 5 A is created in BioRender (Johansson A, 2026; https://BioRender.com/j0ujbky) and licensed under CC BY 4.0.

Finally, the reduction of punished alcohol self-administration seen following *FgfR1* knockdown was replicated in a dose-dependent manner by systemic administration of the FGFR1 inhibitor PD173074, a small brain penetrant molecule previously shown to suppress alcohol intake in mice as well as rats^20,29^ (Fig. 6D; RM ANOVA resistance score: main effect of treatment; F_(2,42)_ = 10.2, *P* = 0.0002; HSD *post-hoc*: 5 mg/kg vs. vehicle: *P* = 0.10; 15 mg/kg vs. vehicle *P* = 0.006; Fig. 6E main effect of treatment Reinforcers; F_(2,42)_ = 11.3, *P* = 0.0001; HSD *post-hoc*: 5 mg/kg vs. vehicle: *P* = 0.2; 15 mg/kg vs. vehicle *P* = 0.002). PD173074 also showed a trend toward reducing unpunished alcohol self-administration, but this effect did not reach statistical significance (F_(2,27)_ = 1.45; *P* = 0.2; Fig. 6F). In addition, PD173074 did not suppress operant responding for a non-drug reinforcer, a highly rewarding saccharin solution, indicating that its effects were behaviorally specific. In fact, PD173074 at 5 mg/kg produced a trend for a modest increase in responding for saccharin, while 15 mg/kg, the dose that robustly suppressed compulsive-like alcohol self-administration, did not affect responding for saccharin compared to vehicle (Supplementary Fig. 10; one way ANOVA: main effect of treatment; F_(2,25)_ = 3.8, *P* = 0.04; HSD *post hoc test*: 5 mg/kg vs. vehicle *P* = 0.23; 15 mg/kg vs. vehicle *P* = 0.58).

Together, these results identify FGFR1 in PKCδ+ neurons as a key regulator of compulsive-like alcohol self-administration and underscore its potential as a candidate therapeutic target.

## Discussion

Understanding the neural mechanisms that promote vulnerability to compulsive alcohol use is essential for developing targeted interventions, yet the cellular pathways governing this behavior remain poorly defined. Here, we identify cell-type-specific transcriptomic alterations within the CeA *PKCδ*+ neurons associated with punishment-resistant alcohol self-administration in rats, a phenotype that captures a core diagnostic feature of human alcohol addiction. These results are consistent with our prior work, which established a causal role for *PKCδ*+ neurons, but importantly extend those findings by uncovering FGFR1 as a potential molecular mechanism and therapeutic target contributing to compulsive-like alcohol self-administration.

Our findings show robust transcriptomic remodeling within *PKCδ*+ neurons in rats exhibiting punishment-resistant self-administration. This is in line with prior observations that CeA *PKCδ*+ neurons are preferentially activated in punishment-resistant rats, and show the highest number of differentially expressed genes during alcohol withdrawal^8,10^. A recent human snRNAseq study also shows that the highest number of differentially expressed genes in the CeA of AUD individuals occur in PENK+ GABAergic neurons^30^. Our data show that *Penk* is predominantly expressed in PKCδ^+^ and DLK1^+^ neurons of rat CeA (see Fig. 2C), suggesting potential convergence on Penk-expressing CeA populations across species. As the human dataset defines a limited number of cell clusters and does not report co-expression with *PKC*δ or *DLK1*, the relationship between these populations remains to be further resolved.

Convergent anatomical and functional evidence suggests that lateral CeA (CeL) PKCδ+ neurons may promote compulsive-like alcohol intake by suppressing CeA outputs that normally support behavioral inhibition to aversive stimuli, potentially via projections to the periaqueductal gray (PAG)^31^. Although the precise CeA → PAG pathway underlying compulsive-like alcohol use remains to be established, this model is consistent with prior findings showing that reduced mPFC→PAG activity promotes punishment-resistant alcohol drinking in mice^9^. CeA-mediated PAG inhibition, combined with weakened cortical excitation, may thus reduce aversive neural signals, thereby facilitating alcohol self-administration despite footshock. Together, these data support a framework in which enhanced CeA PKCδ+ neuron activity, combined with weakened cortical engagement of aversion-processing circuits, diminishes the behavioral impact of punishment and permits compulsive alcohol intake.

Transcriptomic analyses further identified FgfR1 as a potential upstream regulator of multiple gene expression changes within *PKCδ+* neurons. Pathway enrichment showed engagement of canonical FgfR1 signaling cascades, including PI3K/AKT, RAS/MAPK and PLCγ, all of which were enriched in a co-expression module significantly altered in *PKCδ+* neurons. Consistent with this, *FgfR1* knockdown also reduced *Prkcd* mRNA levels. This supports a model in which FgfR1 signaling orchestrates a coordinated transcriptional state that biases *PKCδ+* neurons toward a heightened activity state, in turn promoting compulsive-like alcohol self-administration. We functionally validated the role of this pathway, as cell-type-specific *FgfR1* knockdown selectively reduced punishment-resistant alcohol self-administration without affecting unpunished responding for alcohol. This demonstrates that FgfR1 signaling within PKCδ⁺ neurons is specifically required for compulsive, rather than general alcohol consumption. Punishment-resistant rats had cumulatively consumed more alcohol than punishment-sensitive rats, raising the possibility that their elevated *FgfR1* expression may reflect greater alcohol exposure. Consistent with this interpretation, chronic alcohol exposure has previously been shown to increase *FgfR1* expression^32,33^. However, the association between punishment resistance and *FgfR1* expression remained significant after controlling for alcohol intake, indicating that differences in alcohol exposure alone do not fully account for the observed transcriptional changes. Together, these findings suggest that increased FgfR1 signaling is linked to the compulsive-like drinking phenotype itself and may reflect a combination of pre-existing vulnerability factors and experience-dependent neuroadaptations associated with prolonged alcohol exposure, potentially resulting in a vicious cycle.

These findings extend a growing body of evidence linking the FGF2/FgfR1 pathway to alcohol-related behaviors. Prolonged alcohol exposure increased the expression of both *Fgfr1 and its ligand Fgf2* in the dorsomedial striatum (DMS), together with changes in *Fgf2* and *Fgfr1* promoter methylation, suggesting epigenetic regulation of this pathway^34^. FgfR1 blockade in the DMS has previously been shown to reduce alcohol drinking and preference, reduce operant alcohol self-administration and modulate alcohol-associated dopamine signaling^20^. Conversely, intra-DMS infusion of its ligand FGF2 promoted alcohol drinking and preference^33^. Together, these previous findings support a role for FGF2/FgfR1 signaling in broadly regulating alcohol reward and consumption^29,32^.

Complementing those observations, our present findings identify a more selective role for FgfR1 signaling within *PKCδ* + CeA neurons in controlling punishment-resistant, compulsive-like alcohol intake, without affecting unpunished alcohol consumption. Whereas DMS-associated FgfR1 signaling may more broadly regulate the reinforcing and motivational properties of alcohol, CeA *PKCδ* + FGFR1 signaling appears to specifically regulate compulsive-like alcohol self-administration despite adverse consequences. Together, these findings suggest that FgfR1 signaling synergistically contributes to distinct dimensions of alcohol related behaviors through anatomically and cell-type-specific mechanisms. Thus, targeting FgfR1 activity across these complementary circuits may both reduce alcohol motivation and restore inhibitory control over maladaptive alcohol seeking.

Collectively, these findings have important translational implications. We provide evidence that FgfR1 activity can effectively and dose-dependently be modulated using the orally available, brain-penetrant small molecule FgfR1 inhibitor PD173074. Although less selective and therefore of less utility as a tool for our target validation, the FGFR1-3 inhibitor infigratinib has demonstrated acceptable human safety and tolerability in cholangiocarcinoma and achondroplasia, and has also shown promising activity in a preclinical model of multiple sclerosis^35^. Together, these data suggest that pharmacological modulation of FgfR1 in the central nervous system may be feasible as a therapeutic strategy.

A limitation of the present study is its exclusive focus on male rats. Sex-specific mechanisms likely contribute to punishment-resistant alcohol self-administration, as previous work suggests that this behavior in males predominantly engages reward-related circuits, whereas it relies more on stress-related pathways in females^12^. Supporting this distinction, we have observed that punishment-resistant females do not show the CeA *Prkcd* mRNA increase that is prominent in males, indicating that the molecular underpinnings of compulsive alcohol intake are sex specific. Additional studies are therefore needed to identify female-specific molecular pathways that promote compulsive-like alcohol self-administration. This work is currently ongoing.

In conclusion, our results identify FgfR1 signaling in CeA *PKCδ+* neurons as a key regulator of compulsive-like alcohol self-administration in male rats. By linking cell-type-specific transcriptional programs to circuit-level mechanisms and behavior, this work provides a framework for understanding vulnerability to alcohol addiction and offers a promising avenue for developing therapeutic interventions.

## Methods

### Animals

Adult male Wistar rats (200–225 g; Charles River, Germany: 7-8 weeks at the beginning of the experiments) were housed under a reverse light cycle with ad libitum access to food and water. All procedures adhered to the guidelines of the Swedish National Committee for Animal Research and were approved by the Local Ethics Committee for Animal Care and Use at Linköping University.

### Alcohol self-administration

Alcohol self-administration was conducted in sound-attenuating chambers (30.5 × 29.2 × 24.1 cm; Med Associates Inc., St. Albans, VT). Rats (∼ 10 weeks old) were trained to self-administer 20% alcohol under a fixed ratio 1 (FR1) schedule during 30-min sessions. Once stable responding was achieved on FR1, the reinforcement schedule was switched to a fixed ratio 2 (FR2).

### Footshock-punished alcohol self-administration

Once baseline behavior stabilized under the FR2 schedule, rats (∼ 5 months old) were assessed for compulsive alcohol intake by pairing each alcohol delivery with a mild footshock (0.2 to 0.3 mA). To quantify resistance to punishment, a resistance score was calculated as follows: (number of alcohol deliveries during punishment sessions) / (number of alcohol deliveries during punishment sessions + average deliveries during the final three unpunished sessions. Rats were then categorized as punishment-resistant, punishment-sensitive, or intermediate using *k-means* cluster analysis. Of the 52 rats, 44% (23) were classified as compulsive-like, 34% (18) as non-compulsive, and 22% (11) as intermediate. For snRNA-seq, the 12 rats with the highest resistance scores (RS) and the 12 with the lowest RS were selected (Fig. 1C). For comparison, yoked control rats received the same number and intensity of footshocks as their punishment-resistant counterparts, but these shocks were not contingent on their own behavior.

### Foot shock sensitivity

Footshock delivery began at 0.05 mA and increased by 0.05 mA every 30 s while the animals remained in the operant chamber. Thresholds were determined for the removal of 1, 2, 3, and all 4 paws from the grid.

### Surgery

Surgeries were performed to investigate the causal role of FGFR1 in PKCδ⁺ neurons in compulsive alcohol use, we utilized a transgenic rat line developed in our laboratory that expresses Cre recombinase specifically in PKCδ⁺ neurons^12^. A Cre-dependent AAV vector encoding a *shRNA* targeting *FgfR1* was then injected to achieve selective knockdown of *FgfR1* in these neurons. Animals (∼ 5 months old) were anesthetized with isoflurane (4%, Baxter mixed with air, 400cc/min) and injected with buprenorphine [0.03 mg/kg, subcutaneously (s.c.)] 30 min before surgery to relieve pain. During the surgery, the animal was constantly inhaling isoflurane mixed air (2-3% isoflurane, 300 cc/min). Carprofen (5 mg/kg, s.c.) was injected after surgery and the following day to relieve pain and reduce inflammation. Animals were allowed to recover from surgery for 1 week and were then exposed to the footshock punishment procedure 4 weeks later to allow the viral expression.

For viral microinjections into the CeA, the following stereotaxic coordinates were used: antero-posterior (AP), −2.5 mm; medio-lateral (ML), ±4.5 mm; and dorso-ventral (DV), −8.5 mm. Rats received bilateral infusions of either an *FgfR1* knockdown vector (AAV5-hSyn-cDIO-GFP-miR30-NM_024146_3916_v2; TTTGTTTTTAATATATAGCTAC; titer >2 × 10^13 GC/ml) or a scrambled control vector (AAV5-hSyn-cDIO-GFP-miR30-shRNA; titer >2 × 10^13 GC/ml). A volume of 0.5 μL per side was delivered at a flow rate of 0.1 μL/min, followed by a 5-min diffusion period.

### Generation of shRNA against Fgfr1

We designed shRNAs targeting the 3’ untranslated region (UTR) of rat Fgfr1 (NM_0214146) using SplashRNA (http://splashrna.mskcc.org^36^; which identified five 22-mer oligonucleotides with SplashRNA scores > 1.0 and no matches to mouse or human genes. We chose the sequence with the highest SplashRNA score to generate our construct (NM_024146_3916; TTTGTTTTTAATATATAGCTAC; SplashRNA score: 1.593084004). The selected shRNA and a control shRNA [RM1.1] targeting luciferase were subcloned into an identical miR30 cassette within a Cre-dependent pAAV vector containing a Syn1 promoter and EGFP reporter^37^ to produce AAVs (serotype 5) for microinjection in vivo. AAV plasmids and viruses were generated by VectorBuilder, Inc.

### Systemic PD173074 administration during punished alcohol self-administration

PD173074 (MedChemExpress) was dissolved in a vehicle consisting of 10% DMSO, 40% PEG300, and 5% Tween-80 in saline. Following the footshock-punished self-administration paradigm, rats were categorized as punishment-resistant or punishment-sensitive. PD173074 (0, 5, or 15 mg kg^−1^) was administered intraperitoneally (2 ml kg^−1^) 8 h before each session using a within-subject Latin square design, such that each rat received all three doses in a counterbalanced order^29^. Baseline levels of punished alcohol self-administration were re-established between drug tests across two consecutive sessions. Rats were approximately 5 months old at the time of testing.

### RNAscope in situ hybridization assay

Immediately after the final self-administration session, animals were deeply anesthetized with isoflurane (4% in air, 400 mL/min; Baxter). Adequate anesthesia was confirmed by the absence of the pedal withdrawal reflex. Animals were then rapidly decapitated using a guillotine, and the brains were immediately collected and snap-frozen in dry ice-cooled isopentane. Eighteen-micrometer-thick brain sections were then collected at the CeA level and stored at −80 °C until use. In situ hybridization was performed according to the RNAscope Fluorescent Multiplex Kit v2 User Manual (Advanced Cell Diagnostics, Newark, CA, USA). The *FgfR1* probe (accession no. NM_024146.1) and *Prkcd* probe (accession no. NM_133307.1) were obtained from Advanced Cell Diagnostics. Images were acquired using a Leica STELLARIS 5 confocal microscope equipped with a 40× objective lens.

Quantitative analysis of mRNA expression was conducted using the open-source software QuPath^®^ (v0.51). All RNAscope-labeled images were imported into QuPath^®^, where a machine learning–based classifier was trained for each marker to detect positive cells and subcellular dots (representing probe signals). The same parameter settings were applied across all images for each marker to ensure consistency.

### Tissue collection

Rats (∼ 5 months old) were deeply anesthetized with isoflurane (4% in air, 400 mL/min; Baxter) immediately after the final self-administration session. Adequate anesthesia was confirmed by the absence of the pedal withdrawal reflex. Animals were then rapidly decapitated using a guillotine, and the brains were immediately collected and snap-frozen in dry ice-cooled isopentane and stored at −80 °C until further processing. We sectioned the brains at a cryostat (Leica CM3050s) and collected 300 µm slices from approximately bregma −1.6 mm to −2.8 mm based on the rat brain in stereotaxic coordinates. The CeA tissue was then harvested bilaterally from the brain slices using a 1.5 mm diameter biopsy punch, and tissue blocks were stored at −80 °C.

### Nuclei isolation and snRNA-seq library preparation

Nuclei were isolated using the Chromium Nuclei Isolation Kit (10x Genomics, Pleasanton, CA) following the manufacturer’s protocol, with a lysis time of 6.5 min and one 1-ml wash post the debris removal step. The nuclei were resuspended in a final volume of 50–100 µL at a target concentration of 500–1200 nuclei/µL. Single-nucleus RNA-seq libraries were generated using the Chromium Next GEM Single Cell 3′ Kit v3.1 (10x Genomics), targeting 10,000 nuclei per sample. Libraries were sequenced on the Illumina NovaSeq 6000 system using an S1 flow cell, producing approximately 500 million reads per sample. Library preparation and sequencing were performed at the Genomic Sequencing and Analysis Facility, Center for Biomedical Research Support, The University of Texas at Austin (RRID:SCR_021713).

### snRNA-seq data processing

#### Quality control and clustering

The RNA sequencing data generated in this study have been deposited in the GEO database under accession code GSE310229. The data obtained using 10× Genomics was initially processed using the Cellranger pipeline v-7.1.0. Reads were aligned to the mRatBN7 reference transcriptome and loaded into a Seurat object for further processing. Doublet detection was performed using scDblFinder for each rat separately, and cells predicted to be doublets were filtered. Quality control metrics were calculated and included the number of genes per cell, number of transcripts per cell and percentage of reads mapping to mitochondrial (percent.mt) and ribosomal RNA (percent.ribo). Cells from three rats (M8, M22 & M33) were removed from the analysis as their quality metrics significantly differed from the rest of the samples. For the remaining samples cutoffs were set for the number of genes per cell, percent.mt and percent.ribo at <7000, <5 and <2.5 respectively. Genes mapping to mitochondrial or ribosomal RNA were removed from the dataset for downstream analysis steps. After quality control 393141 cells remained in the dataset (131422 from punishment resistant, 121023 from punishment sensitive, 140696 from yoked controls). The count matrix was split into sample layers and normalized using SCTransform with vars.to.regress set to percent.mt and percent.ribo. The resulting count matrix was then subjected to dimensionality reduction. Principal components were computed using RunPCA with default settings, and RunUMAP was used with the first 50 components and metric set to ‘euclidean’ for visualization of the dimensionality reduction. Unsupervised clustering of the cells was performed using FindNeighbors() on the first 50 PCs, followed by FindClusters() at a resolution of 0.1. FindAllMarkers() was used to detect cluster-specific marker-transcripts, which were then used to annotate the clusters based on established markers. Correction for ambient RNA was performed after annotation of main cell types using decontX from celda, with delta = 3.55, 20. The resulting corrected count matrix was used for all downstream analyses.

#### Subclustering of GABAergic cells

The dataset was subset to only include the cell types labeled as ‘Gabaergic’. This subset was subjected to an additional round of quality control, and was filtered to only include cells with more than 1500 unique genes, number of transcripts between 500 and 20000, less than 1 percent.ribo and less than 3 percent.mt, leaving 121584 cells for further analysis (44152 punishment resistant, 35108 punishment sensitive, 42324 yoked controls). The dataset was split into sample layers, which were then subjected to the same normalization, dimensionality reduction and clustering steps as described for the main cell types, but with a clustering resolution of 0.5. The resulting 39 clusters of presumed gabaergic subtypes were annotated based on differential expression of marker genes.

#### Differential expression analysis

Differential expression analysis of individual transcripts between experimental groups was performed using the R package distinct, which applies hierarchical non-parametric permutation tests to compare the cumulative distribution functions of each sample across groups. This approach is particularly well suited for single-nucleus RNA-sequencing data analyzed across biological replicates (https://doi.org/10.1214/22-AOAS1689). To identify transcripts associated with compulsive-like alcohol drinking independently of foot shock exposure, differential expression analyses were conducted between compulsive and non-compulsive rats, as well as between compulsive and foot shock–yoked rats. Transcripts overlapping between the two analyses were considered to be specifically associated with compulsive-like drinking behavior. Briefly, the dataset containing all gabaergic cells was subset to include compulsive and non-compulsive samples only, and a design matrix containing sample ids and group ids was generated. Using this, distinct_test was run on the “cpm” assay, using gabaergic subtypes as “name_cluster” and sample ids as “name_sample”, column_to_test = 2, min_non_zero_cells = 2000 and setting permutations for when a raw *P* value is <0.001 to 20,000. Genes were considered to be differentially expressed at a globally adjusted *P* value < 0.05.

#### Co-expression network analysis

Weighted gene co-expression network analysis (WGCNA) was performed using hdWGCNA (PMID: 37426759). To aid in the comparison between groups, consensus network analysis was made to construct co-expression networks conserved across all three experimental conditions. This analysis first constructs individual networks for each experimental group, which are then used to compute an integrated network for the whole dataset. An initial filtering of genes was set such that genes that were expressed in less than 5% of all cells were omitted. To reduce the sparsity of the data, metacells were constructed using MetacellsByGroups(), which utilizes the k-Nearest Neighbors algorithm to aggregate small groups of cells originating from the same biological sample. The k-parameter was set to 25, and max-shared was set to 12, grouping by SampleID and Group. TestSoftPowers() with networktype = ‘signed’ was used to find a threshold for the soft power where the co-expression network has a scale-free topology, and the network was then constructed using ConstructNetwork() with soft powers set to 9 for punishment resistant and 8 for the other two groups. Module eigengenes and module connectivity were calculated using ModuleEigengenes() with group.by.vars = ‘SampleID’ and ModuleConnectivity() respectively. The expression of module eigengenes within each cell was added to the Seurat object’s metadata. Differential expression analysis of module eigengenes between groups was performed at the level of all gabaergic cells as well as gabaergic subtypes using FindDMEs() with the Wilcoxon signed-rank test for both punishment-resistant vs sensitive and punishment-resistant vs yoked. Modules expressed in at least 50% of the analyzed cluster, while also exhibiting significant differential expression at an adjusted *P* value of <0.05 in both punishment-resistant vs sensitive and punishment-resistant vs yoked, were selected for further analysis. Functional analysis of modules was performed by manual examination of the gene sets as well as extracting the genes for each module and importing the resulting gene lists into IPA, obtaining canonical pathways that significantly overlap with the module sets, focusing on pathways including the genes with the highest kME ((QIAGEN Inc., https://digitalinsights.qiagen.com/IPA)). Gene onthology enrichment analysis was performed on the top 100 genes of each module based on kME using the databases ‘GO Biological_Process_2025’, ‘GO_Cellular_Component_2025’, ‘GO_Molecular_Function_2025’.

#### Pathway analysis

Pathway analysis of co-expression modules and differentially expressed genes was performed using QIAGEN IPA (QIAGEN Inc., https://digitalinsights.qiagen.com/IPA). For co-expression module analyses, gene lists were provided without additional quantitative values, enabling pathway enrichment analysis without the calculation of z-scores. During enrichment analysis, pathways clearly specific to organs other than the nervous system were excluded. Furthermore, because genes within each co-expression module are expected to be co-regulated and change in the same direction, only pathways for which the constituent genes were predicted to change concordantly were considered. For differentially expressed genes, values for adjusted *P* value and log_2_(fold change) were also included, allowing for the calculation of z-scores.

### Statistical analysis

For statistical analysis of self-administration data across days, repeated-measures analysis of variance (RM ANOVA) was used when the assumption of sphericity was met, as assessed by Mauchly’s test of sphericity. When the sphericity assumption was violated, a linear mixed-effects model with autoregression (AR(1)) covariance structured was used. Levene’s test was used to assess the homogeneity of variances. Two-tailed independent t-tests were used to analyze RNAscope in situ hybridization expression data. *P* values < 0.05 were considered statistically significant. Except for the analysis of sequencing data, all statistical analyses were conducted using statistica^®^ or SPSS^®^.

### Reporting summary

Further information on research design is available in the Nature Portfolio Reporting Summary linked to this article.

## Supplementary information

## Source data

## Data Availability

The RNA sequencing data generated in this study have been deposited in the GEO database under accession code GSE310229 https://www.ncbi.nlm.nih.gov/geo/query/acc.cgi?acc=GSE310229. Source data for all figures is provided in this paper. Source data are provided with this paper.

## Code availability

The code used to perform these analyses is publicly available on GitHub at https://github.com/leohog/Fgfr1_analysis. For package version information, see supplementary data 3.
