# Supplementary material for: Identification and validation of central amygdala FGFR1 as a therapeutic target for alcohol use disorder using single-nucleus sequencing in rats
Source: Nat Commun. 2026 Sep 2;17:9407. doi: 10.1038/s41467-026-77302-9 (PMC13538410; doi:10.1038/s41467-026-77302-9)
Supplement: Supplementary file 1 [file 41467_2026_77302_MOESM1_ESM.pdf]

Supplementary materials

Supplementary Results

Cell-type clustering

Following initial quality control and filtering, a total of 393141 nuclei were retained, with an average of 4261 transcripts and 2123 unique genes expressed per cell (131422 from compulsive-like, 121023 from non-compulsive and 140696 from yoked-control rats). A subset of lower-quality cells containing Hbb-b1 - likely reflecting blood contamination - was also identified. A second round of quality control and filtering was applied to the GABAergic subset, yielding 121,584 high-quality nuclei with an average of 8,231 transcripts and 3,372 unique genes per nucleus (44,152 from compulsive-like, 35,108 from non-compulsive, and 42,324 from yoked controls).

Region specific enrichment of gene expression within CeA

Hierarchical clustering of annotated cell subtypes based on expression of genes previously shown to be enriched in certain parts of the CeA revealed expected regional differences (Fig. 2C). For example, one branch of the dendrogram contained subtypes enriched for MSN markers such as Penk, Six3 and Ppp1r1b as well as genes marking projecting neurons such as Wfs1 and Ptkb2 (9). This group contained Foxp2 expressing neurons identified as cells of the intercalated amygdala (IA) as well as Calcrl/Prkd1-expressing neurons of the lateral part of the CeA (CeL) including Drd2-expressing populations as well as the previously studied CeL Prkcd expressing neurons (48, 51). A cluster of Dlk1 expressing neurons also expressed Ptk2b, Six3 and Penk but lacked expression of Calcrl, Prkd1 and Ppp1r1b. This cluster is likely to represent the CeM Dlk1 neurons recently studied for their role in nausea induced feeding (52), and together with the CeL Prkcd neurons they make up a group of Fgfr1 expressing neurons. Three clusters had specific expression of palliadal markers Lhx8 and Megf10 and were grouped together on the dendrogram with a cluster enriched for Sox6 and Ano4, neither of which is known to be expressed in the CeA. These groups were also separated from the bulk of the nuclei in UMAP space and likely represented accidental captures from nearby structures.

A large group of clusters expressed Zfhx3 and/or Tshz2, shown to be enriched in the CeM (9). Three of these clusters had high expression of Ebf1 and no expression of Tac3, resembling the previously described major source of CeM-projections to the PBN (11). A small cluster of cells expressing Slc17a8 was also present here, potentially representing the VGLUT3-positive cells of the CeA shown to be involved in bitter taste perception (53). Two Tshz2 expressing

clusters also expressed Foxp2, often used as a marker of intercalated cells of the amygdala (IA) together with Tshz1, which they lacked. Furthermore, they expressed Nr2f2 which has been shown to be expressed in cells occupying the transition zone between the medial and lateral subdivisions of the CeA, a transition zone containing the highest density of projections to the lateral hypothalamus (ref). Interestingly, mouse connectivity data suggests lateral hypothalamus does indeed receive projections from Foxp2 expressing neurons of the CeA, indicating that these Foxp2/Nr2f2 neurons might not originate in the IA but rather in the transition zone between lateral and medial CeA (54). However, since we lack spatial information in this dataset, for clarification all clusters with a robust expression of Foxp2 were annotated as Intercalated cells along with genes that differentiated them.

Another group of clusters expressed Lhx6 and Nxph1 and corresponded to a population of neurons previously shown to reside in the MEA, likely representing components of the amygdalar-hypothalamic pathway described to play a role in reproductive behavior (9,55). Two of these clusters also expressed Nr2f2, possibly placing them in the aforementioned transition zone. These clusters also lacked expression of Isl1. In the study that identified Nr2f2 as a marker of the transition zone between lateral and medial CeA, the authors also described Isl1 as being predominantly expressed in the most medial parts of the CeA (54). The clusters in our dataset exhibiting the clearest pattern of high Nr2f2 expression and low Isl1 expression were Dlk+ neurons, Lhx6+ neurons, Vip+ interneurons, VGLUT2+ neurons and the Foxp2+/Tshz1- neurons. Conversely, clusters with high expression of Isl1 but low expression of Nr2f2 included the previously described group of Ebf1+ clusters, VGLUT3+ neurons, Sst+ neurons expressing Nos1 as well as a number of clusters not adhering to previously known types. In UMAP space cells generally clustered according to this convention of Nr2f2/Isl1-ratio, but there were also clusters that did not clearly fall into either category.

One cluster specifically expressed Vipr2, along with Sst and Pdyn. This cluster expressed markers associated with the CeL as well as the CeM and was separated from the large bulk of nuclei in UMAP space. Others have shown Vipr2 to mark a distinct Sst expressing population situated in the medial portion of the CeA, restricted to the posterior and middle parts of the CeA (11). Other clusters that showed unique gene signatures were Drd2 expressing neurons that also express Rai14, small clusters of Mast4 expressing neurons, a small cluster expressing Grm3 and a cluster of Tf/Car2 expressing nuclei. Generally, QC metrics showed similar distributions between clusters apart from the Tf_Car2 cluster which mostly contained low quality cells (Fig S3). Prkcd+ cells of the CeL were the most abundant celltype in all three groups, and the proportions of celltypes were similar among the groups (Supp file 1). For a complete list of differentially expressed genes per subtype, see (Supp file 2).

Supplementary figures

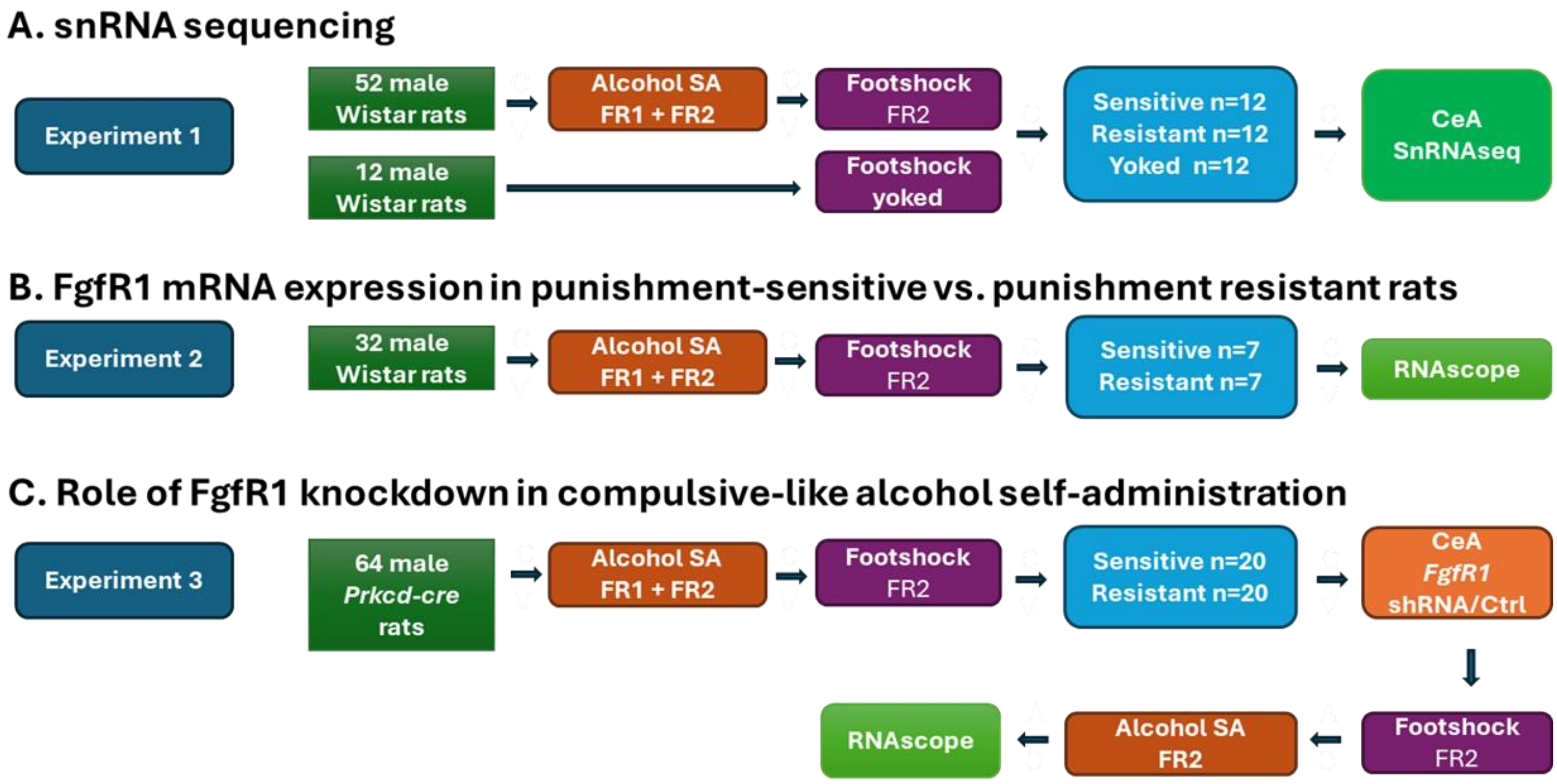

Fig. S1. Experimental timelines

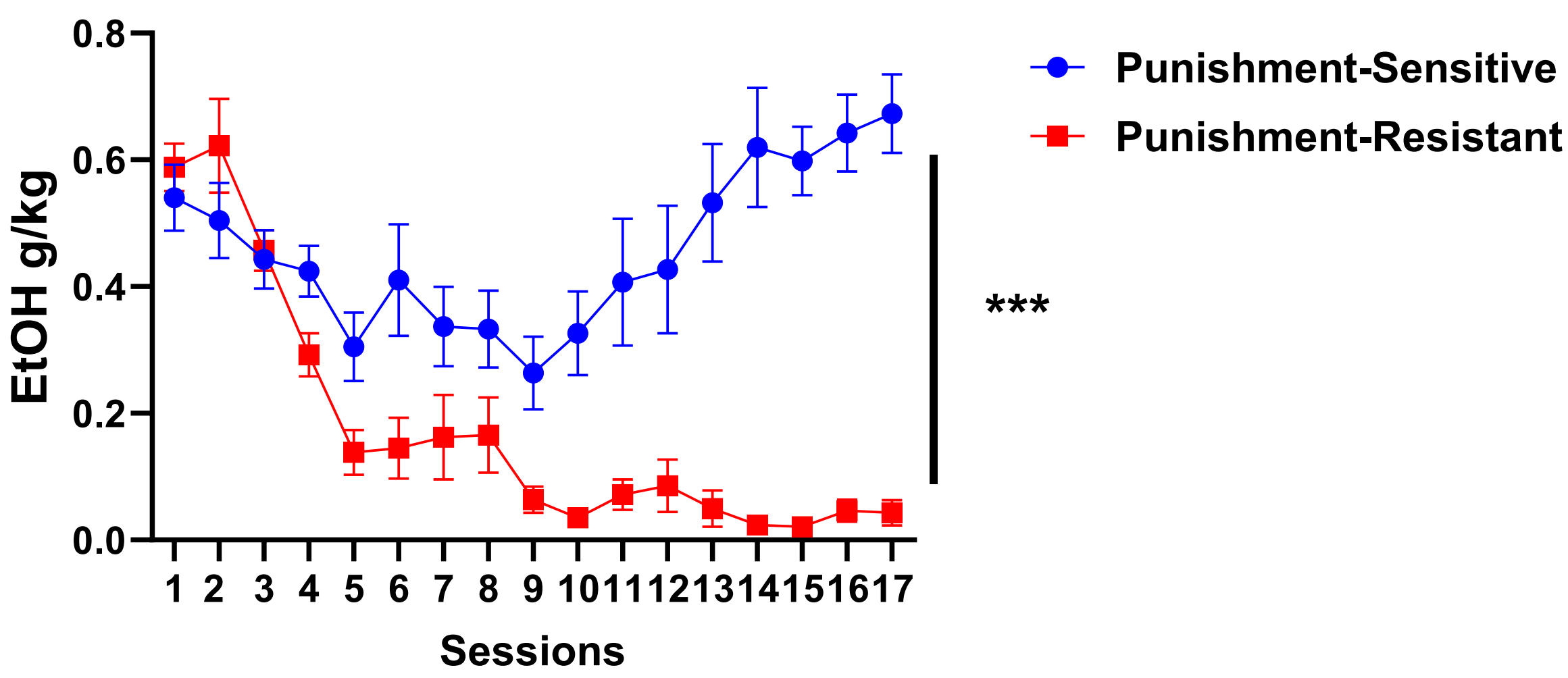

Fig. S2. Average alcohol rewards (g/kg) in punished self-administration. Ethanol intake (g/kg) across punishment sessions in punishment-sensitive (blue, n = 12) and punishment-resistant (red, n = 12) rats. Data are presented as mean ± SEM. A linear mixed-effects model with group, session, and their interaction as fixed effects, rat as a random intercept, and an AR(1) covariance structure revealed significant main effects of group ($F_{(1,\ 91.98)}$ = 24.33, $p < 0.001$) and session ($F_{(1,\ 93.13)}$ = 8.97, p = 0.004), as well as a significant group × session interaction ($F_{(1,\ 93.13)}$ = 33.18, $p < 0.001$). Error bars indicate SEM. *** $p < 0.001$ indicates a significant group × session interaction.

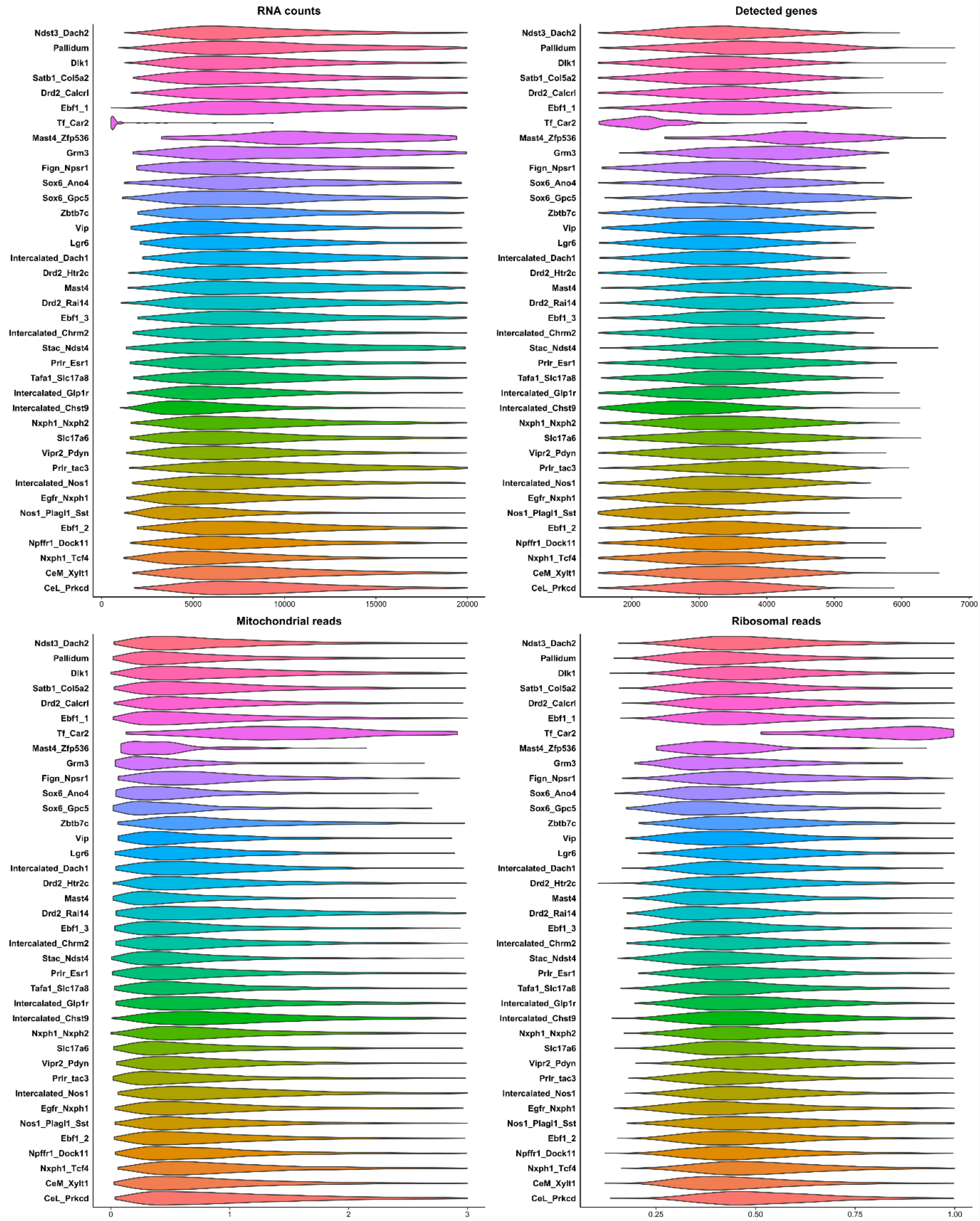

Fig. S3. Quality metrics for GABAergic subtypes. nCount_decontXcounts = number of transcripts per cell, nFeature_decontXcounts = unique transcripts per cell, percent.mt = percentage of transcripts mapping to mitochondrial RNA per cell, percent.ribo = percentage of transcripts mapping to ribosomal RNA per cell.

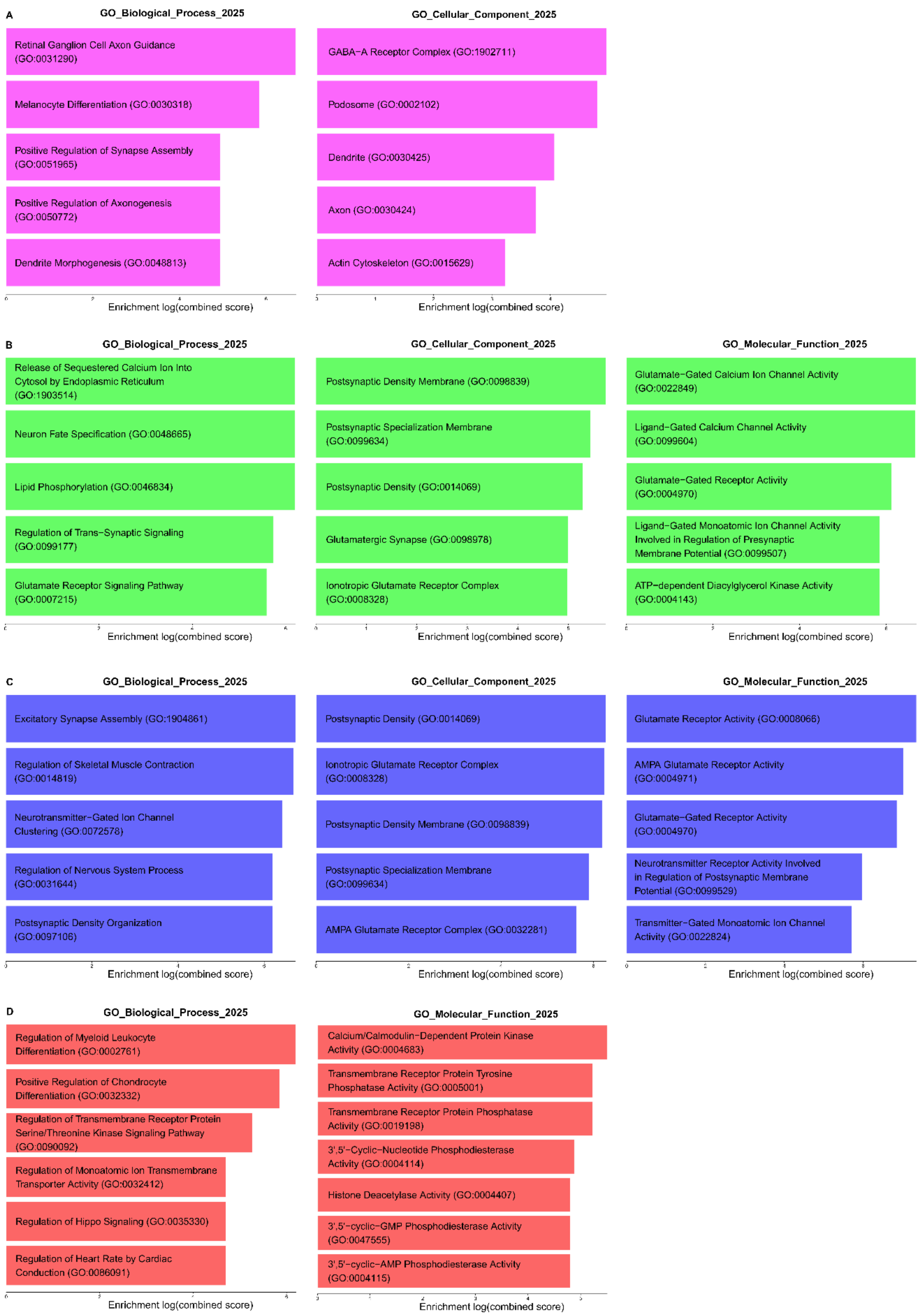

Fig. S4. Gene ontology analysis results from differentially expressed co-expression modules. A. Module 2, B. Module 4, C. Module 5 and D Module 8. P adjusted for multiple comparisons <0.05.

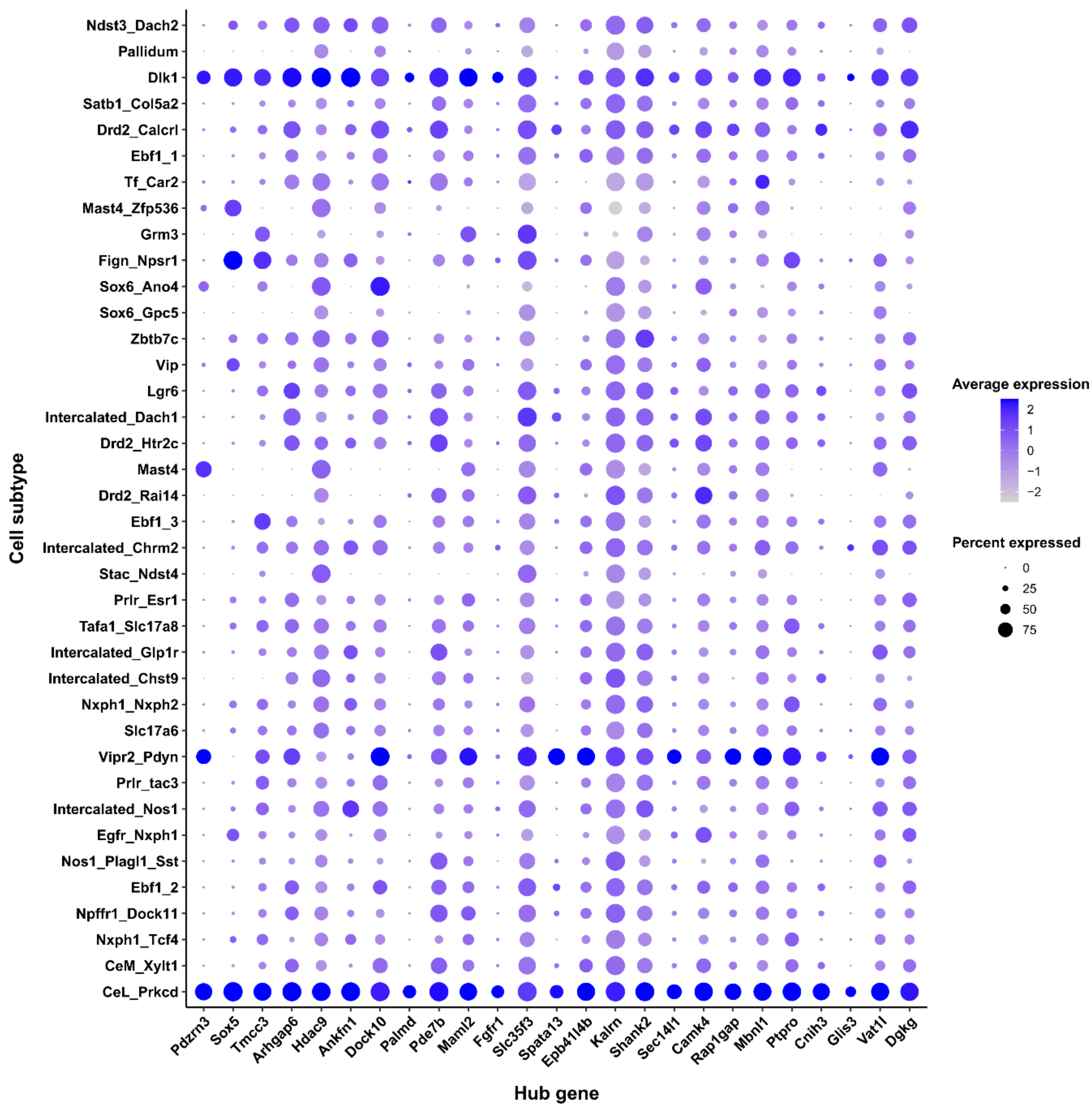

Fig. S5. Expression levels of top 25 hub genes in module 8 across all GABAergic subtypes.

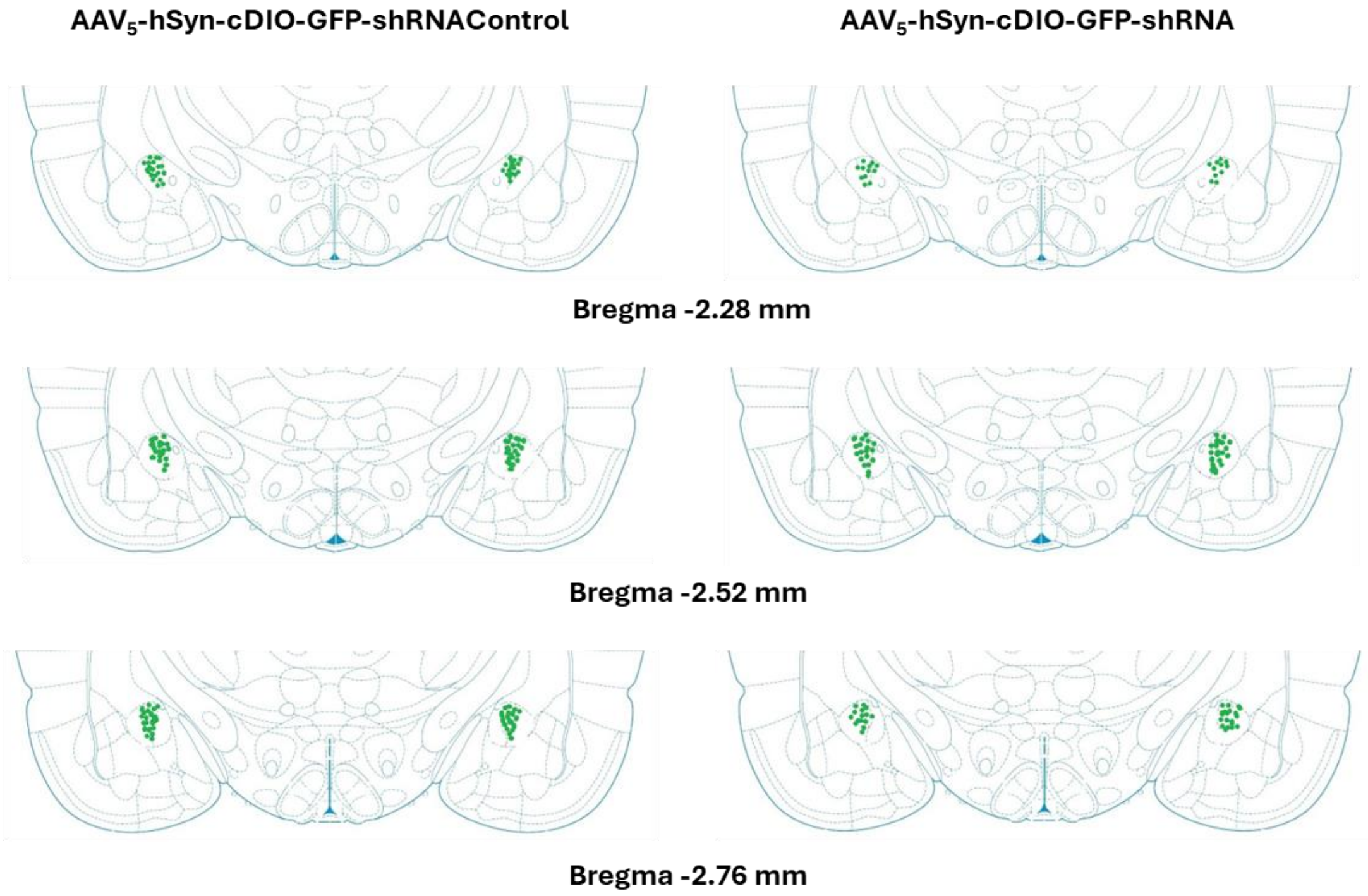

Fig. S6. Representative plots of the spread of the virus injections in the CeA (mm from Bregma).

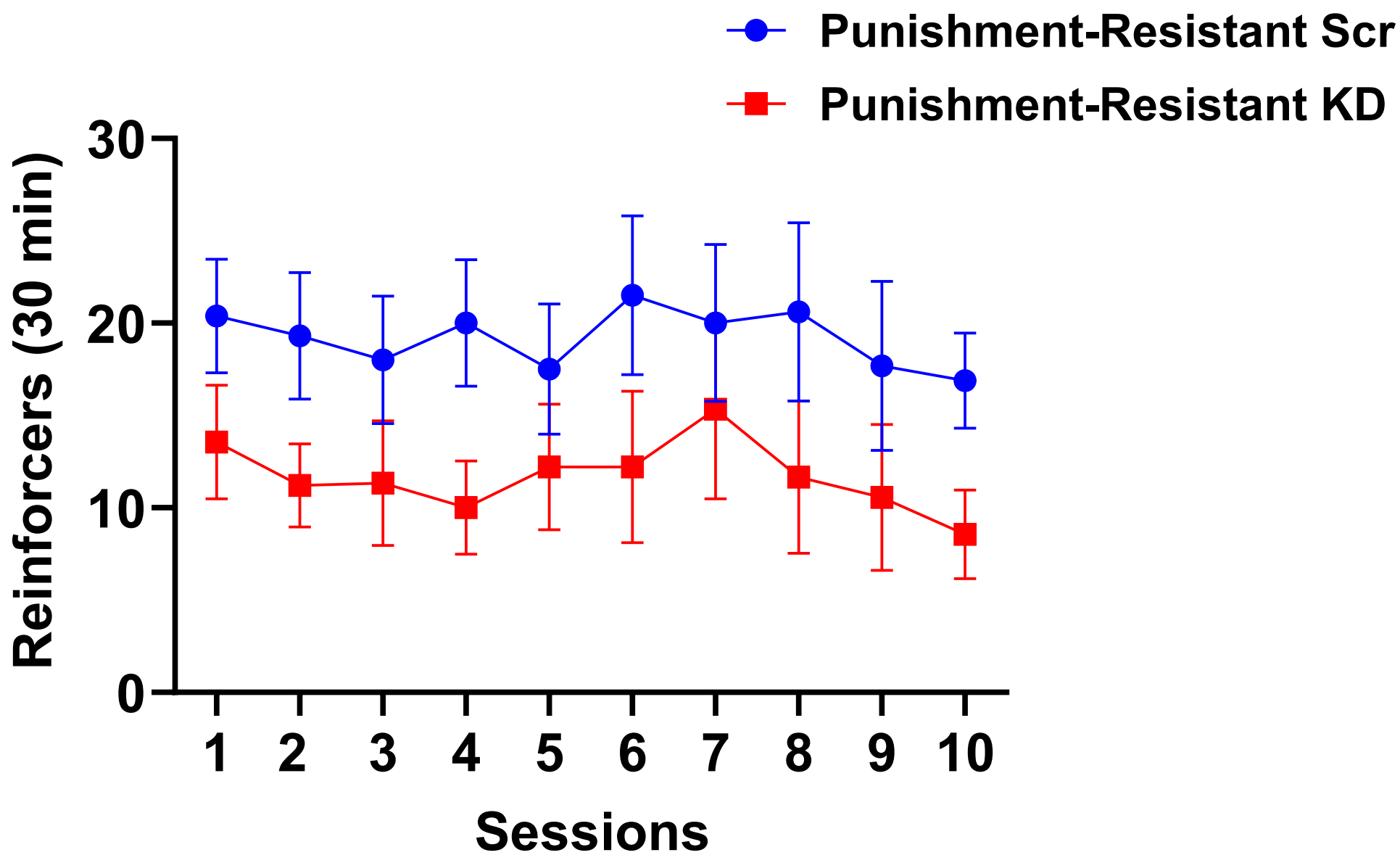

Fig. S7. Effect of FgfR1 KD on punished alcohol self-administration as measured as reward. Alcohol rewards during punished self-administration after knockdown of *Fgfr1* (last 10 days) in punishment-resistant rats expressing a scrambled control vector (punishment-sensitive Scr; blue, n = 10) or Fgfr1 knockdown (Punishment-sensitive KD; red, n = 10). Data are presented as mean ± SEM. A repeated-measures ANOVA revealed a trend toward a main effect of group ($F_{(1,18)} = 3.34$, $p = 0.084$). Scr: Scrambled; KD; knockdown.

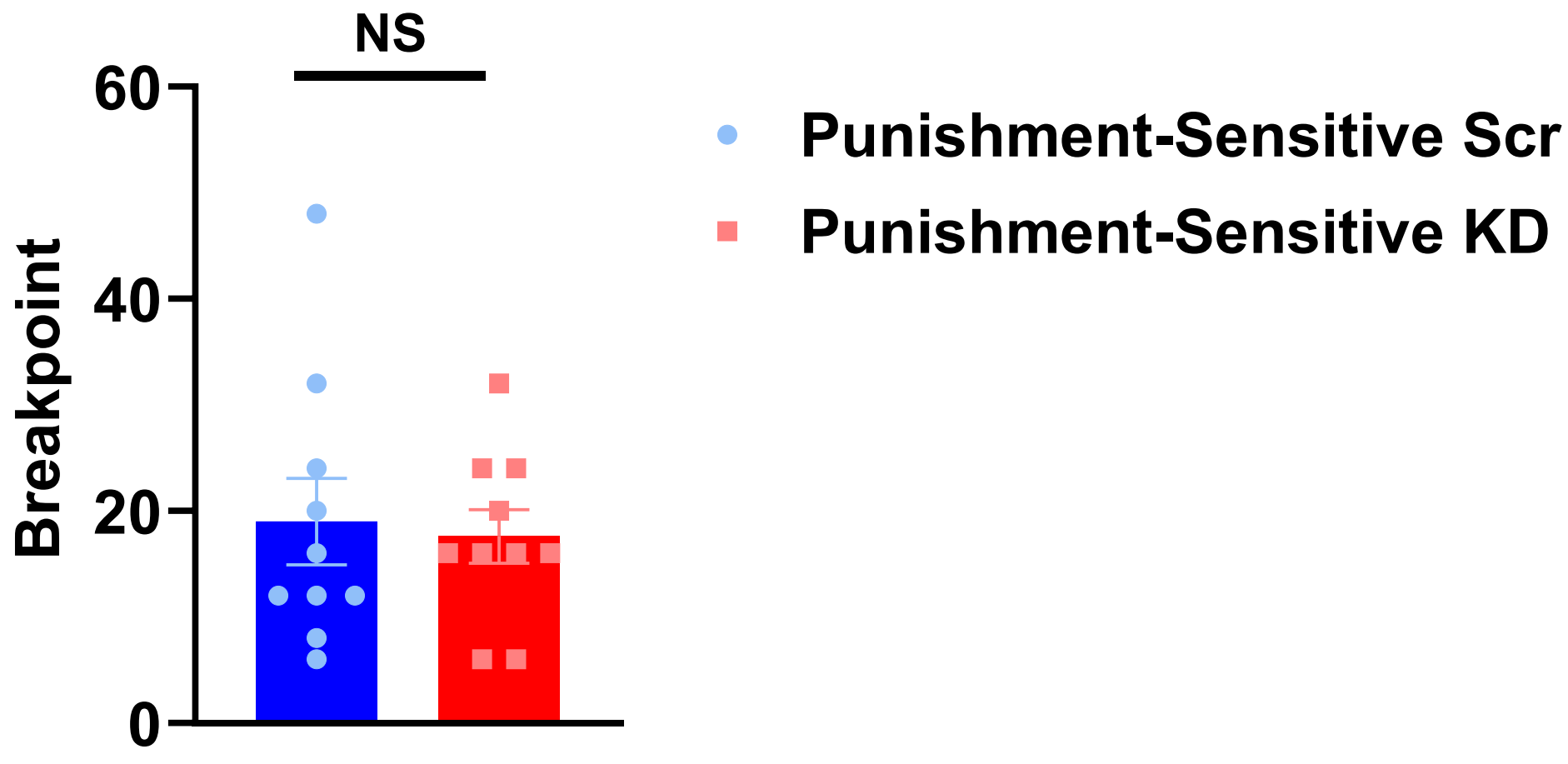

Fig. S8. *FgFR1 KD* does not affect motivation to consume alcohol. Breakpoint obtained during the progressive ratio test in punishment-resistant rats expressing either a scrambled control vector (Punishment-sensitive Scr; blue, n = 10) or *FgfR1* knockdown (Punishment-sensitive KD; red, n = 10) in PKCδ^{+} neurons of the central amygdala. Data are presented as mean ± SEM, with individual animals shown as symbols. An independent-samples, two-tailed t-test revealed no significant difference in breakpoint between groups ($t(18) = 0.29$, $p = 0.77$). NS, not significant; Scr: Scrambled; KD; knockdown.

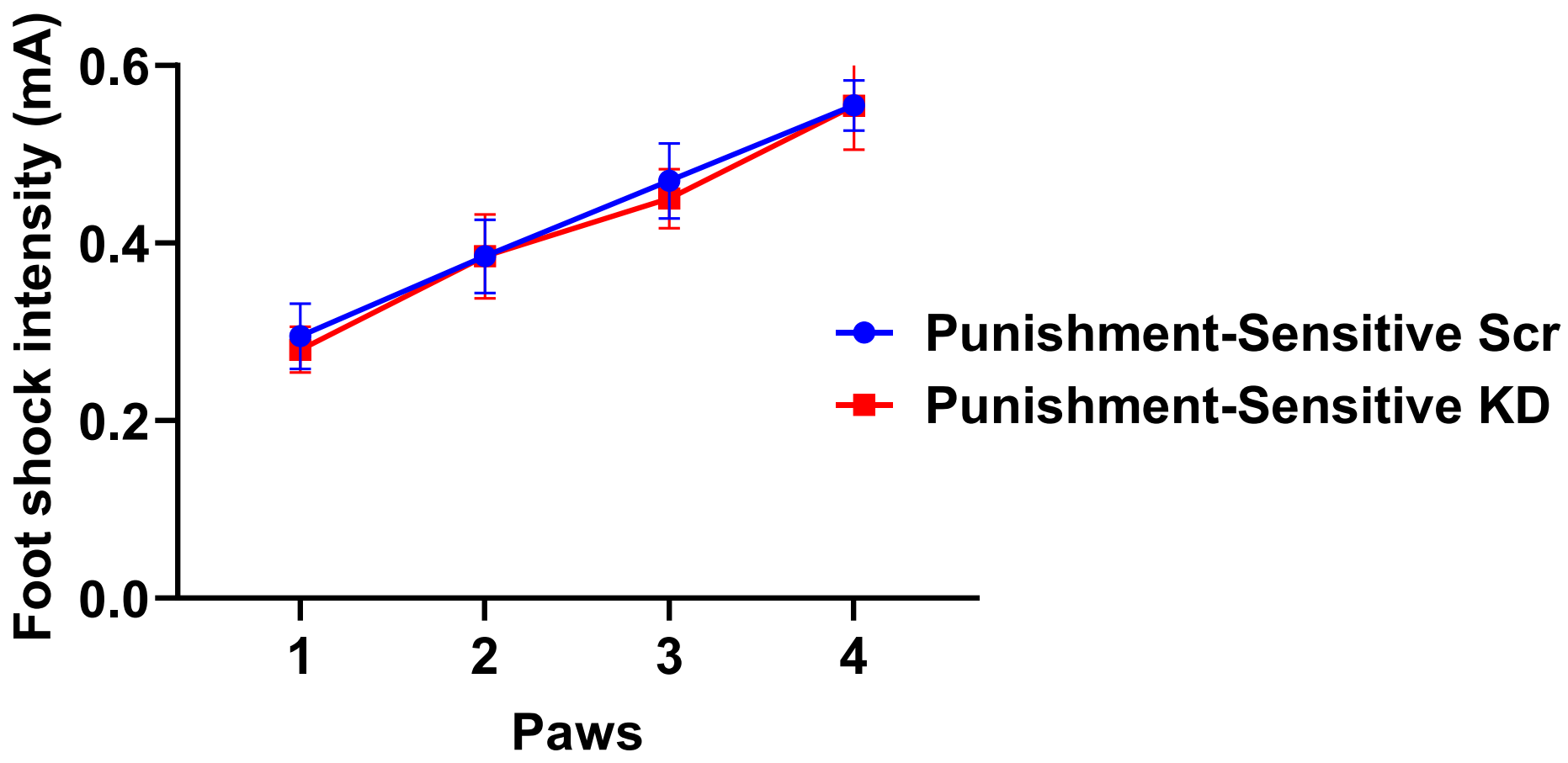

Fig. S9. FgfR1 knockdown did not influence shock sensitivity. RM ANOVA showed no significant effect of group: $F_{(1,54)}$=0.4; P=0.75). Punishment-sensitive Scr are shown in blue (n = 10) and Punishment-sensitive KD are shown in red (n = 10). Data are shown in Mean ± SEM (Scr: Scrambled; KD; knockdown).

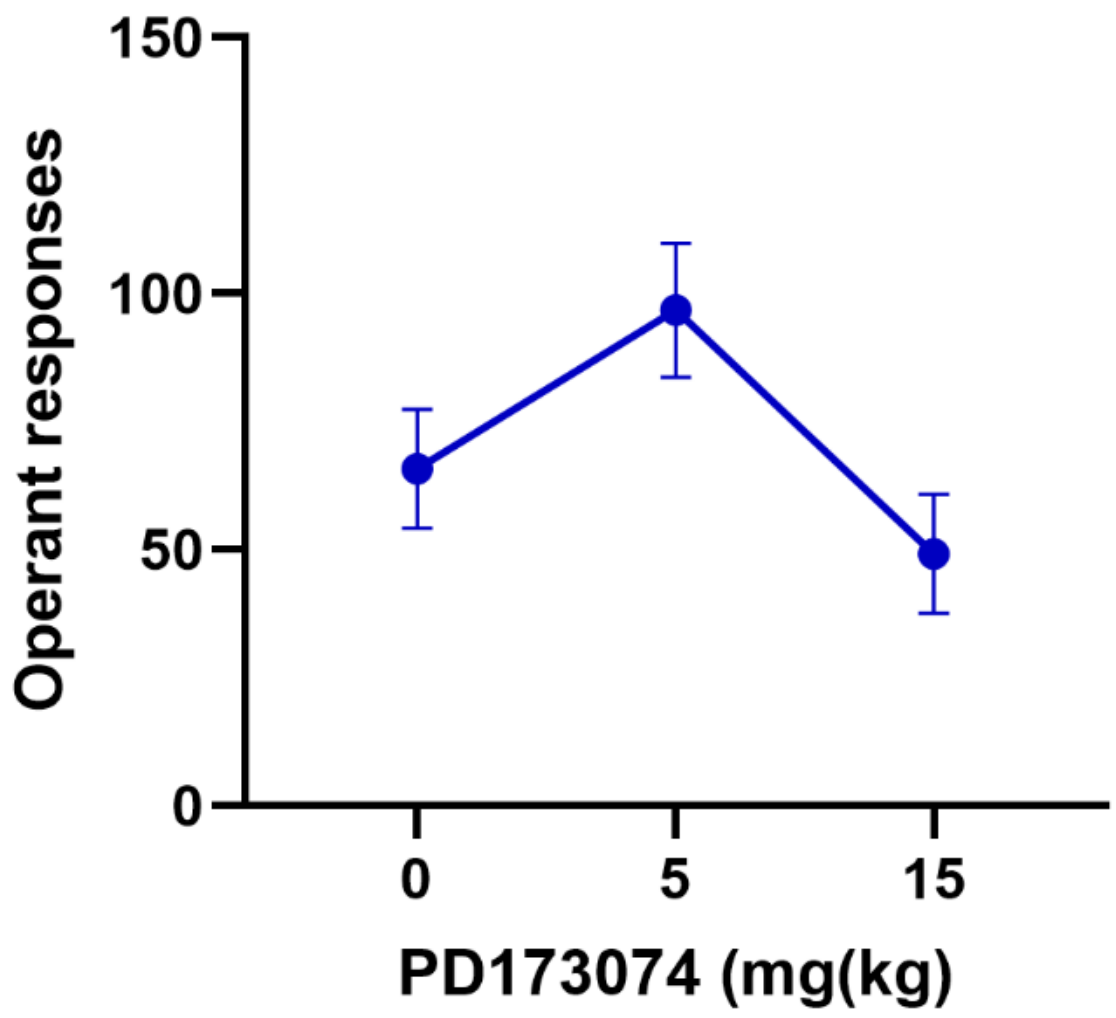

Fig S10. At systemic (i.p.) doses that robustly suppressed compulsive-like alcohol self-administration (main manuscript, Fig. 5E), PD173074 did not suppress operant responding for a non-drug reinforcer, a highly rewarding saccharin solution; in fact, at 5mg/kg, PD173074 produced a trend for an <u>increase</u> in responding for saccharin, while 15mg/kg, the dose that robustly suppressed compulsive-like alcohol self-administration, did not affect responding for saccharin compared to vehicle (one way ANOVA: main effect of treatment; $F_{(2,25)}$=3.8, P=0.04; HSD *post hoc test*: 5mg/kg vs. vehicle P=0.23; 15mg/kg vs. vehicle P=0.58).
