# Supplementary material for: Identification and validation of central amygdala FGFR1 as a therapeutic target for alcohol use disorder using single-nucleus sequencing in rats
Source: Nat Commun. 2026 Sep 2;17:9407. doi: 10.1038/s41467-026-77302-9 (PMC13538410; doi:10.1038/s41467-026-77302-9)
Supplement: Supplementary file 2 [file 41467_2026_77302_MOESM2_ESM.pdf]

nature portfolio

Peer Review File

Identification and validation of central amygdala FGFR1 as a therapeutic target for alcohol use disorder using single nucleus sequencing in rats

Corresponding Author: Dr Markus Heilig

This file contains all reviewer reports in order by version, followed by all author rebuttals in order by version.

Version 0:

Reviewer comments:

Reviewer #1

(Remarks to the Author)
The manuscript of Barbier et al. presents a comprehensive and thoughtfully designed study aimed at identifying molecular mechanisms underlying vulnerability to compulsive-like alcohol use. Using an elegant and translationally relevant model of punishment-resistant alcohol self-administration, the authors combine large-scale single-nucleus RNA sequencing with cell-type-specific and pharmacological manipulations to identify FGFR1 signaling in PKCδ⁺ neurons of the central amygdala as a key regulator of compulsive-like alcohol intake. The study is conceptually strong, with a clear and well-justified progression from behavior to cell-type-specific molecular profiling and causal validation. In particular, the behavioral model is highly compelling, capturing individual differences in aversion-resistant drinking, a core and clinically relevant feature of addiction, and providing a powerful framework for mechanistic dissection. The integration of unbiased transcriptomics with targeted manipulations is executed in a rigorous and convincing manner, resulting in a coherent and biologically meaningful story. Overall, this is an important and timely contribution with clear translational relevance and significant potential impact on the field. However, several points should be addressed.

Major Points
1. A central conceptual issue concerns the interpretation of the molecular differences between punishment-resistant and punishment-sensitive animals. By design, these groups differ not only in their behavioral phenotype, but also in their recent alcohol intake during the punishment phase, with the compulsive group consuming substantially more alcohol. This introduces a potential confound, as the observed transcriptional changes may reflect pre-existing vulnerability mechanisms and/or experience-dependent adaptations induced by higher alcohol exposure. While the authors control for footshock exposure and note relatively modest BAC levels, this specific confounding factor is not explicitly addressed. Importantly, prior work indicates that alcohol exposure itself can alter Fgf2/Fgfr1 expression (Even-Chen et al., 2017; Even-Chen and Barak, 2019; Herburg et al., 2023), further emphasizing this issue. I do not view this as a flaw in the design, as it is a well-known and difficult challenge in such models, but I strongly encourage the authors to explicitly acknowledge this confound, discuss its implications, and more carefully frame their conclusions regarding causality versus consequence.

2. The manuscript cites prior work demonstrating that FGFR1 signaling regulates alcohol-related behaviors, but the relationship to these findings could be more clearly articulated. In particular, previous work (e.g., Grinchii et al., 2023) has shown that systemic FGFR1 inhibition reduces operant alcohol self-administration and alcohol consumption per se, likely via dopaminergic mechanisms. In contrast, the present study shows that FGFR1 manipulation, especially via PKCδ⁺-specific knockdown in the CeA, selectively reduces punishment-resistant (compulsive-like) alcohol intake, without affecting unpunished consumption. This is an important and convincing finding that highlights a circuit- and cell-type-specific role of FGFR1. While this specificity is well presented, it is not sufficiently contextualized within prior literature demonstrating broader effects on general alcohol intake, versus compulsive-like alcohol behaviors, potentially mediated by distinct neural circuits.

3. More broadly, the Discussion would benefit from a more comprehensive integration with the literature on the FGF2–FGFR1 pathway in alcohol-related behaviors. In addition to the studies cited, prior work has demonstrated that FGF2/FGFR1 acts as an endogenous regulator of alcohol consumption and reward (Even-Chen et al., 2017; Even-Chen et al., 2022), and that chronic alcohol exposure can alter Fgf2/Fgfr1 expression, including via epigenetic mechanisms (Herburg

et al., 2023). These findings are highly relevant for two reasons. First, they support the view that the FGF2–FGFR1 pathway is a core biological regulator of alcohol-related behaviors, strengthening the significance of the current findings. Second, they directly relate to the interpretational issue raised above, as they suggest that FGF2–FGFR1 signaling itself may be dynamically regulated by alcohol exposure.

Minor Points
4. Figure quality/ readability
• Fig. 2B – resolution is too low to clearly distinguish subclusters
• Fig. 2C – dendrogram and labels are difficult to read
• Fig. 3C – font size and resolution limit interpretation of gene/module information

Reviewer #2

(Remarks to the Author)
This is an excellent paper in which the authors combine a translationally relevant animal model of individual differences in punishment resistance (modeling alcohol use despite adverse consequences) with state-of-the-art single-nucleus sequencing and bioinformatics, a new transgenic rat (PKCδ-Cre), and chemogenetics to demonstrate a critical role of central amygdala FGFR1 signaling within PKCδ-expressing cells in punishment-resistant rats. The authors further show that an FGFR1 inhibitor (PD173074) decreases alcohol intake in these rats, identifying a potential translational target.

The results are novel, and the methodological and statistical analyses are rigorous. The manuscript is also clearly written. This work makes a significant contribution to addiction and psychiatry, as it represents a rare case—if not the first—in which single-nucleus sequencing findings are used to identify a molecule with a causal role in individual differences in a behavioral phenotype. In contrast, most prior single-cell sequencing studies in psychiatry have been largely descriptive or have identified causal mechanisms at the group level, rather than explaining individual differences.

My main suggestion for the revision is to provide a more complete presentation of the behavioral data to improve transparency and facilitate replication and extension by other investigators. I recommend adding the following data and corresponding analyses to the main text figures:

Figure 1: Add the number of alcohol rewards and g/kg intake for the four behavioral phases shown in the timeline.

Figure 4: Add a timeline and the behavioral data, as described for Figure 1.

Figure 5: Divide this figure into two to depict the independent experiments: (1) FGFR1 knockout in PKCδ-Cre rats and (2) PD173074 systemic injections in wild-type rats. Include timelines and data for all behavioral phases, as well as testing of punished and non-punished responding. If viral knockdown and pharmacological manipulations were also assessed in punishment-sensitive groups, include these data in the main figures to demonstrate behavioral specificity.

Minor comments

1. Title: Revise to emphasize identification of central amygdala FGFR1 in alcohol punishment resistance as the main finding, as prior work has shown that systemic antagonists reduce alcohol intake in rodents.

2. Abstract: State that experiments were performed in male rats.

3. Introduction: Shorten the justification for using only males; it is sufficient to note that ongoing work shows CeA PKCδ is critical for punishment resistance in males but not females. Also clarify what is meant by “GABAergic activity within CeA biases…” in terms of CeA GABA function and punishment resistance.

4. Single-nucleus bioinformatics data in the figures: Increase figure font sizes (≥7), as current text is difficult to read.

Reviewer #3

(Remarks to the Author)
In this article, Barbier, Höglund et al present a series of studies investigating the role of FGFR1 in a rat model of alcohol use disorder. This is an excellent and comprehensive manuscript that combines a highly relevant behavioral model of alcohol use disorder with impressive molecular analyses. The finding that FGFR1 expression is elevated specifically within PKCdelta cells in punishment-resistant rats is a novel and highly relevant discovery. Overall, the data strongly support the authors' claim that FGFR1 in PKCdelta neurons is a key regulator of compulsive-like alcohol self-administration and highlight its potential as a candidate therapeutic target. I am positive about this paper and believe the findings have significant implications for the future treatment of AUD.

Strengths:

- The justification for the exclusive use of male rats in this specific study is well-reasoned and acceptable, given the authors' explanation of sex-specific mechanistic divergence.
- The molecular analysis is exceptionally impressive and commendable. The authors communicate this highly complex dataset very clearly.
- The progression of the data to Figure 5 constitutes an excellent final set of experiments, clearly showing that the behavioral effects of FGFR1 manipulation are specific to punished alcohol seeking.

Weaknesses/comments:
- The inclusion of the yoked group successfully controls for the high number of footshocks, but it does not control for alcohol exposure. The punishment-resistant group ultimately consumes more alcohol than the sensitive group. While the lack of an alcohol-matched control group does not invalidate the dataset, the authors must discuss the alternative explanation that the transcriptomic differences (including FgfR1 upregulation) could be partially driven by heavier alcohol exposure rather than the compulsive phenotype itself.
- Figures 1C and 1D are slightly misleading. To transparently illustrate both the overall distribution of the population and the fact that the extremes of the population split were selected for sequencing, the excluded "intermediate" rats should be included in these graphs (e.g., in a shaded or grey color). Additionally, the authors should clarify in the figure legend whether the density plots in Figure 1B include the excluded intermediate rats.
- The lack of change in sensitivity to footshock in the FGFR1 knockdown rats (Fig. S9) is an important control. However, this also raises the possibility of other mechanisms, such as a generalized reduction in motivation for alcohol. The authors should consider testing this directly or addressing it thoroughly in the discussion.
- The additional control showing that systemic PD173074 injection does not alter saccharin self-administration is a nice addition. However, it is important to know how this systemic administration affects unpunished alcohol self-administration to ensure the effect is specific to the compulsive-like phenotype, as was done for the viral knockdown.
- Figures 2 and 3 represent a wealth of knowledge for people interested in neuronal sub-types in cenetral amygdala, however I found them somewhat difficult to interpret. The authors could revise the labels/legend to better guide the reader.

Version 1:

Reviewer comments:

Reviewer #1

(Remarks to the Author)
The authors have adequately addressed my concerns, and I have no further comments. This is an excellent manuscript that makes an important contribution to the field.

Reviewer #2

(Remarks to the Author)
The authors have done an excellent job addressing both my comments and those of the other reviewers. I have only one minor suggestion for their consideration:

Supplementary Figures and Figure 1G: Please indicate the session duration on the y-axis labels for the behavioral data, as operant responding is a rate measure. Additionally, if possible, please increase the font size of the bioinformatics panels in the supplementary figures, as they are somewhat difficult to read

Reviewer #3

(Remarks to the Author)
I would like to thank the authors for their thorough and thoughtful responses to my critiques. The inclusion of the ANCOVA to address the alcohol exposure confound, the added transparency in Figure 1, and the new progressive-ratio and unpunished self-administration data have significantly strengthened the manuscript. I have no further comments and believe this manuscript is now ready for publication.

Open Access This Peer Review File is licensed under a Creative Commons Attribution 4.0 International License, which permits use, sharing, adaptation, distribution and reproduction in any medium or format, as long as you give appropriate credit to the original author(s) and the source, provide a link to the Creative Commons license, and indicate if changes were made.
In cases where reviewers are anonymous, credit should be given to 'Anonymous Referee' and the source.
The images or other third party material in this Peer Review File are included in the article's Creative Commons license, unless indicated otherwise in a credit line to the material. If material is not included in the article's Creative Commons license and your intended use is not permitted by statutory regulation or exceeds the permitted use, you will need to obtain permission directly from the copyright holder.
To view a copy of this license, visit https://creativecommons.org/licenses/by/4.0/

REVIEWER COMMENTS FGFR1 Nat. Communication

We thank the reviewers for their positive comments and helpful feedback. Changes in the manuscript are highlighted in red. Our point-by-point responses to the reviewers are listed below.

Reviewer #1:

The manuscript of Barbier et al. presents a comprehensive and thoughtfully designed study aimed at identifying molecular mechanisms underlying vulnerability to compulsive-like alcohol use. Using an elegant and translationally relevant model of punishment-resistant alcohol self-administration, the authors combine large-scale single-nucleus RNA sequencing with cell-type-specific and pharmacological manipulations to identify FGFR1 signaling in PKCδ$^+$ neurons of the central amygdala as a key regulator of compulsive-like alcohol intake. The study is conceptually strong, with a clear and well-justified progression from behavior to cell-type-specific molecular profiling and causal validation. In particular, the behavioral model is highly compelling, capturing individual differences in aversion-resistant drinking, a core and clinically relevant feature of addiction, and providing a powerful framework for mechanistic dissection. The integration of unbiased transcriptomics with targeted manipulations is executed in a rigorous and convincing manner, resulting in a coherent and biologically meaningful story. Overall, this is an important and timely contribution with clear translational relevance and significant potential impact on the field. However, several points should be addressed.

Major Points

1. A central conceptual issue concerns the interpretation of the molecular differences between punishment-resistant and punishment-sensitive animals. By design, these groups differ not only in their behavioral phenotype, but also in their recent alcohol intake during the punishment phase, with the compulsive group consuming substantially more alcohol. This introduces a potential confound, as the observed transcriptional changes may reflect pre-existing vulnerability mechanisms and/or experience-dependent adaptations induced by higher alcohol exposure. While the authors control for footshock exposure and note relatively modest BAC levels, this specific confounding factor is not explicitly addressed. Importantly, prior work indicates that alcohol exposure itself can alter Fgf2/Fgfr1 expression (Even-Chen et al., 2017; Even-Chen and Barak, 2019; Herburg et al., 2023), further emphasizing this issue. I do not view this as a flaw in the design, as it is a well-known and difficult challenge in such models, but I strongly encourage the authors to explicitly acknowledge this confound, discuss its implications, and more carefully frame their conclusions regarding causality versus consequence.

We thank the reviewer for this important comment. To address this issue, we performed an ANCOVA using alcohol intake across the 0.2 and 0.25mA punishment sessions as a covariate. This analysis showed that, after controlling for alcohol intake, group remained a robustly significant predictor of FgfR1 expression. Thus, the observed group differences are not fully explained by differences in alcohol consumption alone.

However, our analysis also did show that alcohol intake itself was significantly associated with Fgfr1 expression, consistent with previous reports demonstrating that alcohol exposure can modulate Fgf2/Fgfr1 expression. We have added in the new statistical analysis in the Result part.

We have then revised the Discussion to clarify that, while elevated Fgfr1 expression may reflect both vulnerability- and exposure-related processes, the functional manipulation data support a direct role for FgfR1 signaling in resistance to punishment.

2. The manuscript cites prior work demonstrating that FGFR1 signaling regulates alcohol-related behaviors, but the relationship to these findings could be more clearly articulated. In particular, previous work (e.g., Grinchii et al., 2023) has shown that systemic FGFR1 inhibition reduces operant alcohol self-administration and alcohol consumption per se, likely via dopaminergic mechanisms. In contrast, the present study shows that FGFR1 manipulation, especially via PKCδ^{+}-specific knockdown in the CeA, selectively reduces punishment-resistant (compulsive-like) alcohol intake, without affecting unpunished consumption. This is an important and convincing finding that highlights a circuit- and cell-type-specific role of FGFR1. While this specificity is well presented, it is not sufficiently contextualized within prior literature demonstrating broader effects on general alcohol intake, versus compulsive-like alcohol behaviors, potentially mediated by distinct neural circuits.

We have now incorporated and discussed the findings from Grinchii et al. (2023) in the revised discussion. Importantly, prior studies demonstrated that systemic FGFR1 inhibition reduces general alcohol self-administration and alcohol consumption, likely through broader dopaminergic mechanisms. In contrast, our findings show that PKCδ^{+} cell-specific FGFR1 KD in the CeA selectively reduces punishment-resistant alcohol self-administration without affecting unpunished alcohol intake. We agree that this distinction is important, as it suggests that FGFR1 signaling may contribute to distinct aspects of alcohol-related behaviors through different neural circuits and cell populations, potentially acting in parallel and synergistically. We have revised the text to better contextualize our findings within broader literature and to emphasize the potentially specialized role of CeA PKCδ^{+} FGFR1 signaling in compulsive-like alcohol self-administration.

3. More broadly, the Discussion would benefit from a more comprehensive integration with the literature on the FGF2–FGFR1 pathway in alcohol-related behaviors. In addition to the studies cited, prior work has demonstrated that FGF2/FGFR1 acts as an endogenous regulator of alcohol consumption and reward (Even-Chen et al., 2017; Even-Chen et al., 2022), and that chronic alcohol exposure can alter Fgf2/Fgfr1 expression, including via epigenetic mechanisms (Herburg et al., 2023). These findings are highly relevant for two reasons. First, they support the view that the FGF2–FGFR1 pathway is a core biological regulator of alcohol-related behaviors, strengthening the significance of the current findings. Second, they directly relate to the interpretational issue raised above, as they suggest that FGF2–FGFR1 signaling itself may be dynamically regulated by alcohol exposure.

We have expanded the Discussion to more comprehensively integrate the literature on the FGF2-FgfR1 pathway in alcohol-related behaviors. We agree that these findings are highly relevant to the interpretation of our results. On one hand, they strengthen the significance of our findings by further supporting a central role for FGF2-FGFR1 signaling in alcohol-related behaviors. On the other hand, they reinforce the possibility that the transcriptional differences observed in punishment-resistant animals may reflect both pre-existing vulnerability mechanisms and neuroadaptations induced by greater alcohol exposure. We have revised the Discussion accordingly to better contextualize our findings within the broader literature and to more carefully address the bidirectional relationship between alcohol exposure and FGF2-FGFR1 signaling.

Minor Points

4. Figure quality/ readability

• Fig. 2B – resolution is too low to clearly distinguish subclusters

We have now increased the resolution for Fig. 2B.

• Fig. 2C – dendrogram and labels are difficult to read

We have increased the font size to improve readability.

• Fig. 3C – font size and resolution limit interpretation of gene/module information

We have increased both font size and figure resolution.

Reviewer #2 (Remarks to the Author):

This is an excellent paper in which the authors combine a translationally relevant animal model of individual differences in punishment resistance (modeling alcohol use despite adverse consequences) with state-of-the-art single-nucleus sequencing and bioinformatics, a new transgenic rat (PKCδ-Cre), and chemogenetics to demonstrate a critical role of central amygdala FGFR1 signaling within PKCδ-expressing cells in punishment-resistant rats. The authors further show that an FGFR1 inhibitor (PD173074) decreases alcohol intake in these rats, identifying a potential translational target.

The results are novel, and the methodological and statistical analyses are rigorous. The manuscript is also clearly written. This work makes a significant contribution to addiction and psychiatry, as it represents a rare case—if not the first—in which single-nucleus sequencing findings are used to identify a molecule with a causal role in individual differences in a behavioral phenotype. In contrast, most prior single-cell sequencing studies in psychiatry have been largely descriptive or have identified causal mechanisms at the group level, rather than explaining individual differences.

My main suggestion for the revision is to provide a more complete presentation of the behavioral data to improve transparency and facilitate replication and extension by other investigators. I recommend adding the following data and corresponding analyses to the main text figures:

We thank the reviewer for his valuable suggestion. In response, we have revised the presentation of the behavioral data to provide a more complete and transparent characterization of the behavioral phenotypes. Specifically, we have incorporated the additional data and corresponding analyses suggested by the reviewer into the main figures. We believe these additions improve the rigor and interpretability of the study. Specifically:

Figure 1: Add the number of alcohol rewards and g/kg intake for the four behavioral phases shown in the timeline.

We have now added the number of alcohol rewards and g/kg intake for the four behavioral phases (Fig. 1B and 1C)

Figure 4: Add a timeline and the behavioral data, as described for Figure 1.

We have now added a timeline and associated behavioral data.

Figure 5: Divide this figure into two to depict the independent experiments: (1) FGFR1 knockout in PKCδ-Cre rats and (2) PD173074 systemic injections in wild-type rats. Include timelines and data for all behavioral phases, as well as testing of punished and non-punished responding. If viral knockdown and pharmacological manipulations were also assessed in punishment-sensitive groups, include these data in the main figures to demonstrate behavioral specificity.

We have reorganized the original Figure 5 into two separate figures to clearly distinguish the two independent experimental approaches. Revised Figure 5 now presents the effects of Fgfr1 knockdown in PKCδ-Cre rats, whereas revised Figure 6 presents the effects of systemic administration of the FGFR1 antagonist PD173074 in wild-type rats. For both figures, we now include detailed experimental timelines and behavioral data from all relevant phases of the procedure, including measures of both punished and non-punished responding. In addition, we have included the corresponding data from punishment-sensitive animals in the main figures to demonstrate the behavioral specificity of the viral knockdown and pharmacological

manipulations (Fig. 5D and E). We believe that these revisions improve the clarity of data presentation.

Minor comments

1. Title: Revise to emphasize identification of central amygdala FGFR1 in alcohol punishment resistance as the main finding, as prior work has shown that systemic antagonists reduce alcohol intake in rodents.

To make this point, the title has been revised to “Identification and validation of central amygdala FGFR1 as a therapeutic target for alcohol use disorder using single nucleus sequencing in rat”

2. Abstract: State that experiments were performed in male rats.

We have added this (in a manner we hope reflects what is transparently explained later, i.e. that the massive sequencing was carried it balanced groups of males and females, and that it was only after obtaining those results that we realized the mechanistic downstream experiments would have to be carried out in males and females separately).

3. Introduction: Shorten the justification for using only males; it is sufficient to note that ongoing work shows CeA PKCδ is critical for punishment resistance in males but not females. Also clarify what is meant by “GABAergic activity within CeA biases…” in terms of CeA GABA function and punishment resistance.

We have shortened this part.

4. Single-nucleus bioinformatics data in the figures: Increase figure font sizes (≥7), as current text is difficult to read.

The font size has been increased.

Reviewer #3 (Remarks to the Author):

In this article, Barbier, Höglund et al present a series of studies investigating the role of FGFR1 in a rat model of alcohol use disorder. This is an excellent and comprehensive manuscript that combines a highly relevant behavioral model of alcohol use disorder with impressive molecular analyses. The finding that FGFR1 expression is elevated specifically within PKCdelta cells in punishment-resistant rats is a novel and highly relevant discovery. Overall, the data strongly support the authors' claim that FGFR1 in PKCdelta neurons is a key regulator of compulsive-like alcohol self-administration and highlight its potential as a candidate therapeutic target. I am positive about this paper and believe the findings have significant implications for the future treatment of AUD.

Strengths:

- The justification for the exclusive use of male rats in this specific study is well-reasoned and acceptable, given the authors' explanation of sex-specific mechanistic divergence.
- The molecular analysis is exceptionally impressive and commendable. The authors communicate this highly complex dataset very clearly.

- The progression of the data to Figure 5 constitutes an excellent final set of experiments,

clearly showing that the behavioral effects of FGFR1 manipulation are specific to punished alcohol seeking.

Weaknesses / comments:

- The inclusion of the yoked group successfully controls for the high number of footshocks, but it does not control for alcohol exposure. The punishment-resistant group ultimately consumes more alcohol than the sensitive group. While the lack of an alcohol-matched control group does not invalidate the dataset, the authors must discuss the alternative explanation that the transcriptomic differences (including FgfR1 upregulation) could be partially driven by heavier alcohol exposure rather than the compulsive phenotype itself.

We agree with the reviewer, and this is also a point made by Revier 1. As stated in the response to that reviewer, to address this issue, we performed an ANCOVA using alcohol intake across the 0.2 and 0.25mA punishment sessions as a covariate. This showed that, after controlling for alcohol intake, group remained a robustly significant predictor of FgfR1 expression. Thus, the observed group differences are not fully explained by differences in alcohol consumption alone.

However, our analysis also did show that alcohol intake itself was significantly associated with Fgfr1 expression, consistent with previous reports demonstrating that alcohol exposure can modulate Fgf2/Fgfr1 expression. We have added in the new statistical analysis in the Result part.

We have then revised the Discussion to clarify that, while elevated Fgfr1 expression may reflect both vulnerability- and exposure-related processes, the functional manipulation data support a direct role for FgfR1 signaling in resistance to punishment.

- Figures 1C and 1D are slightly misleading. To transparently illustrate both the overall distribution of the population and the fact that the extremes of the population split were selected for sequencing, the excluded "intermediate" rats should be included in these graphs (e.g., in a shaded or grey color). Additionally, the authors should clarify in the figure legend whether the density plots in Figure 1B include the excluded intermediate rats.

We thank the reviewer for this helpful suggestion. To improve transparency, we have revised Fig. 1 to include the animals with intermediate punishment sensitivity that were not selected for RNA sequencing (Fig. 1B-E). These animals are now shown in grey. This presentation more accurately reflects the underlying population structure. In addition, we have clarified in the Figure 1 legend that the density plots shown in Fig 1B (now Fig. 1D) were generated using the entire behavioral cohort, including the intermediate animals that were not selected for sequencing. Thus, the density distributions represent the full population, whereas only the extreme phenotypes were subsequently used for RNA-sequencing analyses (Fig. F-G).

- The lack of change in sensitivity to footshock in the FGFR1 knockdown rats (Fig. S9) is an important control. However, this also raises the possibility of other mechanisms, such as a generalized reduction in motivation for alcohol. The authors should consider testing this directly or addressing it thoroughly in the discussion.

We thank the reviewer for this important point. To address the possibility that the effects of FGFR1 knockdown reflect a generalized reduction in motivation for alcohol rather than a specific effect on compulsive alcohol seeking, we assessed motivation using a progressive-ratio schedule of reinforcement. FGFR1 knockdown did not significantly alter breakpoint under

the progressive-ratio schedule, indicating that motivation to obtain alcohol was not affected by the manipulation. These findings, together with the absence of changes in footshock sensitivity (Fig. S9), support the conclusion that FGFR1 knockdown specifically reduces compulsive alcohol-seeking behavior rather than producing a generalized decrease in alcohol motivation or altered sensitivity to punishment. We have now included these results (Supplementary Fig. S8).

- The additional control showing that systemic PD173074 injection does not alter saccharin self-administration is a nice addition. However, it is important to know how this systemic administration affects unpunished alcohol self-administration to ensure the effect is specific to the compulsive-like phenotype, as was done for the viral knockdown.

We agree with the reviewer that it is important to determine whether the effects of systemic PD173074 administration are specific to compulsive-like alcohol seeking or reflect a more general effect on alcohol-directed behavior. In response, we have now included data examining the effects of PD173074 on unpunished alcohol self-administration. Although PD173074 produced a numerical reduction in unpunished alcohol responding, this effect did not reach statistical significance ($F_{(2,27)} = 1.45$, $p = 0.25$; Fig. 6F). In contrast, PD173074 significantly reduced punished responding in compulsive animals. Interestingly, previous studies by Barak and colleagues reported that FGFR1 antagonism with PD173074 reduces alcohol consumption and operant alcohol self-administration. While our data show a similar directional effect, the effect did not reach statistical significance in our study, likely due in part to the substantial inter-individual variability observed in unpunished alcohol self-administration. Therefore, although our findings are not inconsistent with the previous literature, they support a relative preference for a significant effect of PD173074 on punished over baseline alcohol self-administration under the conditions tested.

- Figures 2 and 3 represent a wealth of knowledge for people interested in neuronal sub-types in central amygdala, however I found them somewhat difficult to interpret. The authors could revise the labels/legend to better guide the reader.

We thank the reviewer for the suggestion and have now revise the labels/legend.

REVIEWERS' COMMENTS

We thank the reviewers for their careful evaluation of our manuscript and their positive comments. Our point-by-point responses to the reviewers are listed below.

Reviewer #1 (Remarks to the Author):

The authors have adequately addressed my concerns, and I have no further comments. This is an excellent manuscript that makes an important contribution to the field.

We are pleased that the reviewer found our responses and revisions to have adequately addressed their concerns.

Reviewer #2 (Remarks to the Author):

The authors have done an excellent job addressing both my comments and those of the other reviewers. I have only one minor suggestion for their consideration:

Supplementary Figures and Figure 1G: Please indicate the session duration on the y-axis labels for the behavioral data, as operant responding is a rate measure. Additionally, if possible, please increase the font size of the bioinformatics panels in the supplementary figures, as they are somewhat difficult to read.

We are pleased that the reviewer found our responses and revisions to have adequately addressed their concerns. We have now indicated the session duration on the y-axis labels for the behavioral data. We have also increased the font size of the bioinformatics panels.

Reviewer #3 (Remarks to the Author):

I would like to thank the authors for their thorough and thoughtful responses to my critiques. The inclusion of the ANCOVA to address the alcohol exposure confound, the added transparency in Figure 1, and the new progressive-ratio and unpunished self-administration data have significantly strengthened the manuscript. I have no further comments and believe this manuscript is now ready for publication.

We are pleased that the additional analyses and experiments addressed the reviewer’s concerns and strengthens the manuscript.
