# Supplementary material for: Identification and validation of central amygdala FGFR1 as a therapeutic target for alcohol use disorder using single-nucleus sequencing in rats
Source: Nat Commun. 2026 Sep 2;17:9407. doi: 10.1038/s41467-026-77302-9 (PMC13538410; doi:10.1038/s41467-026-77302-9)
Supplement: Supplementary file 3 [file 41467_2026_77302_MOESM3_ESM.pdf]

Description of Additional Supplementary Files

Supplementary Data 1. Canonical pathways in module 8 identified by Ingenuity Pathway

Supplementary Data 2. Canonical pathways in differentially expressed genes in PKCδ$^+$ neurons identified by Ingenuity Pathway Analysis.

Supplementary Data 3. Packages used for snRNAseq data analysis and version information-
