# Supplementary material for: Identification and validation of central amygdala FGFR1 as a therapeutic target for alcohol use disorder using single-nucleus sequencing in rats
Source: Nat Commun. 2026 Sep 2;17:9407. doi: 10.1038/s41467-026-77302-9 (PMC13538410; doi:10.1038/s41467-026-77302-9)
Supplement: Supplementary file 4 [file 41467_2026_77302_MOESM5_ESM.pdf]

nature portfolio

Corresponding author(s): NCOMMS-26-028234A Markus Heilig

Last updated by author(s): Jul 21, 2026

Reporting Summary

Nature Portfolio wishes to improve the reproducibility of the work that we publish. This form provides structure for consistency and transparency in reporting. For further information on Nature Portfolio policies, see our Editorial Policies and the Editorial Policy Checklist.

Statistics

For all statistical analyses, confirm that the following items are present in the figure legend, table legend, main text, or Methods section.

n/a	Confirmed	
☐	☒	The exact sample size (*n*) for each experimental group/condition, given as a discrete number and unit of measurement
☐	☒	A statement on whether measurements were taken from distinct samples or whether the same sample was measured repeatedly
☐	☒	The statistical test(s) used AND whether they are one- or two-sided<br>*Only common tests should be described solely by name; describe more complex techniques in the Methods section.*
☐	☒	A description of all covariates tested
☐	☒	A description of any assumptions or corrections, such as tests of normality and adjustment for multiple comparisons
☐	☒	A full description of the statistical parameters including central tendency (e.g. means) or other basic estimates (e.g. regression coefficient) AND variation (e.g. standard deviation) or associated estimates of uncertainty (e.g. confidence intervals)
☐	☒	For null hypothesis testing, the test statistic (e.g. *F*, *t*, *r*) with confidence intervals, effect sizes, degrees of freedom and *P* value noted<br>*Give P values as exact values whenever suitable.*
☒	☐	For Bayesian analysis, information on the choice of priors and Markov chain Monte Carlo settings
☒	☐	For hierarchical and complex designs, identification of the appropriate level for tests and full reporting of outcomes
☒	☐	Estimates of effect sizes (e.g. Cohen's *d*, Pearson's *r*), indicating how they were calculated

Our web collection on statistics for biologists contains articles on many of the points above.

Software and code

Policy information about availability of computer code

Data collection	Med associates for collection of self-administration data.
Data analysis	Qupath (v0.51) for analysis of RNAscope data. Cellranger pipeline v-7.1.0. Seurat 5.3.0. hdWGCNA 0.4.06. Celda 1.18.2. R 4.3.3. Distinct 1.14.5. Statistica 13. The code used to perform snRNAseq analyses is publicly available on GitHub at https://github.com/leohog/Fgfr1_analysis.

For manuscripts utilizing custom algorithms or software that are central to the research but not yet described in published literature, software must be made available to editors and reviewers. We strongly encourage code deposition in a community repository (e.g. GitHub). See the Nature Portfolio guidelines for submitting code & software for further information.

Data

Policy information about availability of data

All manuscripts must include a data availability statement. This statement should provide the following information, where applicable:

- Accession codes, unique identifiers, or web links for publicly available datasets
- A description of any restrictions on data availability
- For clinical datasets or third party data, please ensure that the statement adheres to our policy

The RNA sequencing data generated in this study have been deposited in the GEO database under accession code GSE310229 https://www.ncbi.nlm.nih.gov/geo/query/acc.cgi?acc=GSE310229.

Research involving human participants, their data, or biological material

Policy information about studies with human participants or human data. See also policy information about sex, gender (identity/presentation), and sexual orientation and race, ethnicity and racism.

Reporting on sex and gender	N/A
Reporting on race, ethnicity, or other socially relevant groupings	N/A
Population characteristics	N/A
Recruitment	N/A
Ethics oversight	N/A

Note that full information on the approval of the study protocol must also be provided in the manuscript.

Field-specific reporting

Please select the one below that is the best fit for your research. If you are not sure, read the appropriate sections before making your selection.

- [x] Life sciences
- [ ] Behavioural & social sciences
- [ ] Ecological, evolutionary & environmental sciences

For a reference copy of the document with all sections, see nature.com/documents/nr-reporting-summary-flat.pdf

Life sciences study design

All studies must disclose on these points even when the disclosure is negative.

Sample size	12 animals per group fpr snRNAseq. 20 animals per group for Fgfr1-knockdown. 15-22 animals per group for Fgfr1-inhibition. Sample sizes were based on prior experiments in which meaningful effect sizes were detected using the same or lower sample sizes. In particular for the key snRNA-seq experiments, sample sizes widely exceeded those used in prior work of similar nature
Data exclusions	Cells from three rats were excluded from snRNA-seq data analysis based on quality metrics (M8, M22, M33). No samples were excluded from behavioral data.
Replication	Several of the experiments, such as population distribution and identification of the subpopulation of rats showing punishment resistant alcohol self-administration, were in themselves replications of prior experiments. Molecular experiments ensured reproducibility in multiple ways. First, RNA-seq differential gene expression screen was based on unusually large groups to ensure statistical robustness. Primary bioinformatic analysis relied on gene co-expression networks rather than individual transcripts, which are more prone to random variation. Differential expression of target transcripts implied by the RNA-seq analysis was independently confirmed using RNA-scope.
Randomization	Group allocation was made in such a way as to balance the main covariate (punished self-administration at baseline) between the groups.
Blinding	Sequencing was performed in a different lab by people not aware of groups. Analysis of RNAscope data was performed in a blinded way. Behavioral data was obtained automatically.

Reporting for specific materials, systems and methods

We require information from authors about some types of materials, experimental systems and methods used in many studies. Here, indicate whether each material, system or method listed is relevant to your study. If you are not sure if a list item applies to your research, read the appropriate section before selecting a response.

Materials & experimental systems

n/a	Involved in the study	
x		Antibodies
x		Eukaryotic cell lines
x		Palaeontology and archaeology
	x	Animals and other organisms
x		Clinical data
x		Dual use research of concern
x		Plants

Methods

n/a	Involved in the study	
x		ChIP-seq
x		Flow cytometry
x		MRI-based neuroimaging

Animals and other research organisms

Policy information about studies involving animals; ARRIVE guidelines recommended for reporting animal research, and Sex and Gender in Research

Laboratory animals	Wistar rat & transgenic cre-rat on Wistar background
Wild animals	N/A
Reporting on sex	Only male rats were used in this study.
Field-collected samples	N/A
Ethics oversight	All procedures adhered to the guidelines of the Swedish National Committee for Animal Research and were approved by the Local Ethics Committee for Animal Care and Use at Linköping University.

Note that full information on the approval of the study protocol must also be provided in the manuscript.

Plants

Seed stocks	N/A
Novel plant genotypes	N/A
Authentication	N/A
